# Molecular mechanisms of seed dormancy release in *Paeonia lactiflora* revealed through transcriptomic and metabolomic analysis

**DOI:** 10.1186/s12870-025-07636-x

**Published:** 2026-04-16

**Authors:** Yingtong Mu, Kefan Cao, Jingshi Lu, Junjie Wang, Xiaojie Li, Xiaoming Zhang

**Affiliations:** College of Grassland Science/Key Laboratory of Grassland Resources of Ministry of Education, Inner Mongolia Agricultural University, Hohhot, 010018 China

**Keywords:** *Paeonia lactiflora*, Seed dormancy release, Cold stratification, Metabolomics, Transcriptomics, Hormone regulation

## Abstract

**Background:**

*Paeonia lactiflora* Pall., a perennial plant with medicinal and ornamental value, exhibits a typical "double dormancy" characteristic in its seeds, which significantly limits large-scale cultivation. This study combines metabolomics and transcriptomics to explore the molecular mechanisms of dormancy release and germination in *Paeonia lactiflora* seeds during warm-cold stratification, focusing on hormonal regulation, metabolic pathway alterations, and gene expression changes.

**Methods:**

*Paeonia lactiflora* seeds were subjected to stratification for 0, 28, 55, and 80 days (T0, T1, T2, T3). Endogenous hormones (ABA, GA₃, IAA) and sugars (sucrose, glucose, fructose) were quantified using high-performance liquid chromatography coupled with mass spectrometry (HPLC–MS). Nutrient contents and enzyme activities were measured using commercial kits (Solarbio), following the instructions and using standard reagents for quantification. RNA sequencing was performed for transcriptomic analysis, with differential gene expression (DEG) analysis conducted using DESeq2. Gene co-expression networks were built using weighted gene co-expression network analysis (WGCNA) to identify key regulatory modules.

**Results:**

Significant changes in hormone and nutrient contents were observed during stratification. During the warm stratification phase (T0–45 days, 20 °C), ABA (abscisic acid) levels were dominant, while during the cold stratification phase (45–80 days, 4 °C), the seed’s hormonal composition underwent significant changes. ABA levels decreased from 72.54 ng/g at T0 to 1.49 ng/g at T2, GA₃ increased from 0.45 ng/g at T0 to 1.41 ng/g at T1, and IAA levels significantly increased from 4.32 ng/g at T0 to 70.09 ng/g at T1. Sugar levels showed a downward trend, with fructose content decreasing from 22.34% at T0 to 7.31% at T3. Starch content significantly decreased from 40.13% at T0 to 15.34% at T3. Enzyme activities of α-amylase and β-amylase peaked at 0.2267 U/mg and 0.3410 U/mg at T2, respectively. Transcriptomic analysis yielded 83.82 GB of high-quality clean data, identifying 83,082 differentially expressed genes (DEGs). DEG analysis revealed 11,045 DEGs during embryo axis growth (T0–T3), 10,042 DEGs during epicotyl elongation, and 923 DEGs common across all stages. WGCNA analysis identified the black, cyan, and turquoise modules as key regulatory modules related to hormonal regulation and nutrient mobilization. Pathway enrichment analysis showed that DEGs were significantly involved in metabolic pathways, including starch and sucrose metabolism, hormone signaling pathways (IAA, GA, ABA), and oxidative phosphorylation.

**Supplementary Information:**

The online version contains supplementary material available at 10.1186/s12870-025-07636-x.

## Introduction

*Paeonia lactiflora Pall.*, a perennial plant with both medicinal and ornamental value, is widely distributed across temperate regions of China, Russia, Europe, and North America [1]. Its roots serve as the primary source of the traditional Chinese medicine “Chi Shao,” which is known for its heat-clearing, blood-cooling, anti-inflammatory, and analgesic properties. The plant is rich in flavonoids, polysaccharides, and other bioactive compounds, exhibiting pharmacological effects such as anti-fatigue and blood sugar-lowering properties [2, 3]. In recent years, *P. lactiflora* has attracted increasing attention due to its considerable potential in medicinal development and industrial application. However, large-scale cultivation of *P. lactiflora* is severely hindered by its characteristic double dormancy of both the hypocotyl and epicotyl. Specifically, seed germination requires a two-phase dormancy release: the elongation of the hypocotyl (radicle) must first occur under warm stratification, followed by epicotyl (shoot) growth triggered by cold stratification. This entire process generally spans a prolonged winter period, resulting in an extended propagation cycle and low seedling emergence rate, which poses a major obstacle to the commercial cultivation of *P. lactiflora* [4].

Plant hormones play a crucial role in regulating seed dormancy and germination [5]. Among them, abscisic acid (ABA) acts as a negative regulator, playing a pivotal role in inducing and maintaining dormancy, while cytokinins (CKs) promote cell division and bud differentiation, showing a positive effect in breaking the dormancy of the apical and embryo axes. Synthetic cytokinins, such as 6-benzylaminopurine (6-BA), have been widely used to break dormancy and promote growth in various plants, including *Danfeng* seeds and apple axillary buds. Additionally, CK degradation enzymes (CKXs) and ABA metabolic enzymes (e.g., CYP707A) have been confirmed to closely regulate the homeostasis of hormone concentrations during seed dormancy [6].

Apart from hormonal signals, nutrient regulation also plays a key role in seed dormancy release. Several studies have shown that carbohydrates, proteins, and their degradation products not only serve as energy and structural sources but also contribute to signaling regulation, promoting embryo activation and cell activity [7]. In various plants, the dynamic accumulation and mobilization of nutrients are highly correlated with the transition of seeds from dormancy to germination, suggesting a synergistic regulatory mechanism with hormonal signals. Furthermore, secondary metabolites, such as flavonoids, have been found to participate in the maintenance and release of dormancy, potentially influencing cell differentiation through regulation of antioxidant status or interaction with transcription factors. For example, in *Polygonatum cyrtonema*, seeds exhibit a similar “embryo dormancy” phenomenon, where the root can grow normally, but the shoot axis remains dormant. 6-BA treatment can effectively induce germination, whereas GA₃ has a limited effect, suggesting that different hormonal pathways may have divergent effects on shoot activation [8, 9].

In this study, seeds of *P. lactiflora* were used as experimental materials, and a warm–cold stratification system was established to simulate the natural dormancy release process. Given the double dormancy of both the hypocotyl and epicotyl in *P. lactiflora*, warm stratification primarily breaks hypocotyl (radicle) dormancy, while cold stratification releases epicotyl (shoot) dormancy. By integrating targeted metabolomic analysis of endogenous phytohormones, determination of nutrient contents, key enzyme activity assays, and transcriptome sequencing, we systematically investigated the dynamic changes in hormone levels, metabolic pathway reprogramming, and expression patterns of key regulatory genes during dormancy release. The results identified epicotyl elongation as a critical physiological phase for dormancy termination. This study aims to uncover the molecular regulatory mechanisms underlying double dormancy release in *P. lactiflora* seeds, providing theoretical support for dormancy alleviation in *P. lactiflora* and other species exhibiting similar hypocotyl–epicotyl dormancy, and promoting the development of improved propagation and efficient seedling production systems.

## Materials and methods

### Plant materials and treatments

Seeds of the medicinal plant *P. lactiflora* Pall. were used in this study. Mature seeds were collected from Duolun County, Inner Mongolia, a region known as the “Hometown of Chishao.” After collection, seeds were thoroughly cleaned and air-dried in the shade. A random sample of 200 seeds was subjected to TTC (triphenyl tetrazolium chloride) viability testing, and seeds with viability greater than 95% were selected for subsequent experiments.

### Experimental design for root emergence and shoot emergence, and morphological observation

The selected seeds were imbibed in distilled water for 48 h in darkness at 20 °C, with the water changed every 8 h. After imbibition, the seeds were mixed with river sand that had been sterilized by moist heat at 121 °C for 30 min, using a volume ratio of 1:3 (seed to sand). The moisture content was adjusted to 20–30% of field capacity. The mixture was placed into polypropylene turnover boxes (26 cm × 18 cm × 7 cm) and incubated in complete darkness at five different constant temperatures (5, 10, 15, 20, and 25 °C ± 0.5 °C) for 50 days. To maintain adequate moisture, distilled water was added every 2–3 days. Each temperature treatment included three biological replicates with 90 seeds per replicate.

At the end of the incubation, the number of seeds with radicle lengths greater than or equal to half the seed length and the actual radicle lengths (measured with a 0.01 mm vernier caliper) were recorded. The rooting rate was calculated as follows: $$\begin{aligned} \mathrm{Rooting}\;\mathrm{rate}\;(\%)\:=\:&(\mathrm{Number}\;\mathrm{of}\;\mathrm{seeds}\;\mathrm{with}\;\mathrm{root}\;\mathrm{emergence}/\\&\mathrm{Total}\;\mathrm{number}\;\mathrm{of}\;\mathrm{seeds}\;\mathrm{sown})\:\times\:100\% \end{aligned}$$ 

Subsequently, the germinated seeds from the 20 °C treatment were divided into three groups based on radicle length: 0–2 cm, 2–4 cm, and 4–6 cm. For each group, 90 seeds were selected and subjected to cold stratification at 2, 4, or 6 °C (± 0.5 °C) in darkness for 50 days using moist sand. Water was sprayed weekly to maintain moisture. Each “radicle length × temperature” combination included three replicates.

Shoot emergence was defined by the appearance of cotyledons, and the emergence rate was calculated as: $$\begin{aligned} \mathrm{Emergence}\;\mathrm{rate}\;(\%)\;=\;&(\mathrm{Number}\;\mathrm{of}\;\mathrm{seedlings}\;\mathrm{emerged}\;/\\&\;\mathrm{Number}\;\mathrm{of}\;\mathrm{seeds}\;\mathrm{tested})\;\times\;100\% \end{aligned}$$ 

During both the rooting and shoot emergence stages, morphological observations were conducted every five days, and the observations continued for a total of 100 days.

### Targeted metabolomics of plant hormones and determination of physiological indicators

Based on the results of the germination experiments, four representative time points during stratification were selected: 0 days (dry seeds before stratification), 28 days (approximately 50% radicle protrusion), 55 days (approximately 50% of seeds with 4–5 cm radicle length and enlarged buds), and 80 days (approximately 50% of seeds with epicotyl elongation). These time points were designated as T0, T1, T2, and T3, respectively, for the analysis of plant hormones, sugars, and other physiological parameters.

Endogenous hormones (ABA, GA₃, and IAA) and soluble sugars (sucrose, glucose, and fructose) were quantified using targeted metabolomics. The analysis was performed using a high-performance liquid chromatography–electrospray ionization tandem mass spectrometry system (HPLC–ESI–MS; Agilent 1200 UHPLC/6460 QQQ, USA). The chromatographic separation was carried out on an Agilent Zorbax XDB C18 column (150 mm × 2.1 mm, 3.5 μm particle size). The mobile phases consisted of 0.1% formic acid in water (A) and methanol (B), with a flow rate of 0.3 mL/min. The gradient elution program was as follows: 60%A/40%B for the first 1.5 min, switching to 100%B at 6.5 min, and returning to initial conditions (60%A/40%B) within the next 5 min. Hormone and sugar concentrations were quantified using external standards and expressed as ng/g fresh weight.

In addition, to comprehensively evaluate nutrient mobilization and enzymatic activity during seed dormancy release, the following physiological and biochemical indicators were determined:

- Nutrient contents: soluble sugars (BC0035), fructose (BC0275), starch (BC0705), soluble proteins (BC0325), total lipids (BC0515), and proline (BC0290).
- Enzyme activities: superoxide dismutase (SOD, BC0175), peroxidase (POD, BC0195), catalase (CAT, BC0205), α-amylase (BC0405), β-amylase (BC0430), acid phosphatase (BC0925), and protease (BC0500).

All assay kits were purchased from Solarbio (Beijing, China) and used according to the manufacturer’s instructions. Each measurement was conducted in three biological replicates, and results were expressed on a fresh weight basis.

### RNA extraction and transcriptome sequencing

A total of 12 samples were collected for transcriptome sequencing. Total RNA was extracted using the CTAB method (Zhang et al., 2017), and RNA quality was assessed using a NanoDrop spectrophotometer, agarose gel electrophoresis, and an Agilent 2100 Bioanalyzer. Libraries were constructed using the NEBNext® Ultra™ RNA Library Prep Kit (NEB, USA) and sequenced on the Illumina NovaSeq platform (Novogene, Beijing, China) with 2 × 150 bp paired-end reads.

Raw FASTQ files were processed using FastQC and fastp (v0.23.4) to remove adapter sequences, low-quality reads, and reads containing ambiguous bases (N), resulting in high-quality clean reads. Clean reads from all 12 samples were pooled and assembled de novo using Trinity (v2.15.1) in reference-free mode to obtain a non-redundant transcript set. Coding sequences (CDSs) were predicted using TransDecoder, and functional annotation was performed by Diamond/BLASTX searches against NR, Swiss-Prot, KEGG, and GO databases.

Transcript abundance for each sample was estimated using Salmon (v1.10.0) with quasi-mapping to the assembled transcriptome. Transcript- and unigene-level TPM values were calculated and further summarized into gene-level FPKM values for downstream differential expression analysis.

### Differential expression analysis

Differential expression analysis was performed using DESeq2 software to compare gene expression between three stages of treatment. Benjamini–Hochberg method was used for multiple testing correction, and a p-value < 0.05 was considered statistically significant. Differentially expressed genes (DEGs) were selected with the following criteria: mean FPKM ≥ 1, |log₂(FoldChange)|≥ 1, and p-value ≤ 0.01. K-means clustering was performed on all DEGs, and the resulting expression clusters were analyzed for functional enrichment using the KEGG database. Pathways with a p-value ≤ 0.01 were considered significantly enriched.

### Co-expression network construction

Co-expression networks of transcription factors and DEGs were constructed using the WGCNA package to identify relevant regulatory modules. The network results were visualized using Cytoscape v3.5.1.

### Quantitative real-time PCR validation

To validate the reliability of the transcriptome data, 12 representative genes involved in hormone regulation, sugar metabolism, and lipid transformation were selected for qRT-PCR validation based on the hub genes and DEGs identified through WGCNA analysis. RNA extraction, cDNA synthesis, and amplification were conducted as described by Zhang et al. (2017), with amplification performed using the Bio-Rad CFX96 real-time PCR system (USA). Relative expression levels were calculated using the 2^–ΔΔCt method, with three biological replicates for each sample. Primer information is provided in Additional file 1.

### Statistical analysis

All physiological indices, including starch content, fructose content, lipid and protein content, acid phosphatase activity, protease activity, α-amylase and β-amylase activity, and hormone data, were presented as mean ± standard error. Duncan's multiple range test was used to evaluate statistical significance (*p* < 0.05), with different letters indicating significant differences. Bar graphs were constructed using GraphPad Prism 7.0 software (San Diego, USA).

## Results

### Temperature response characteristics of the hypocotyl and epicotyl dormancy release in *P. lactiflora* seeds

**Fig. 1:**
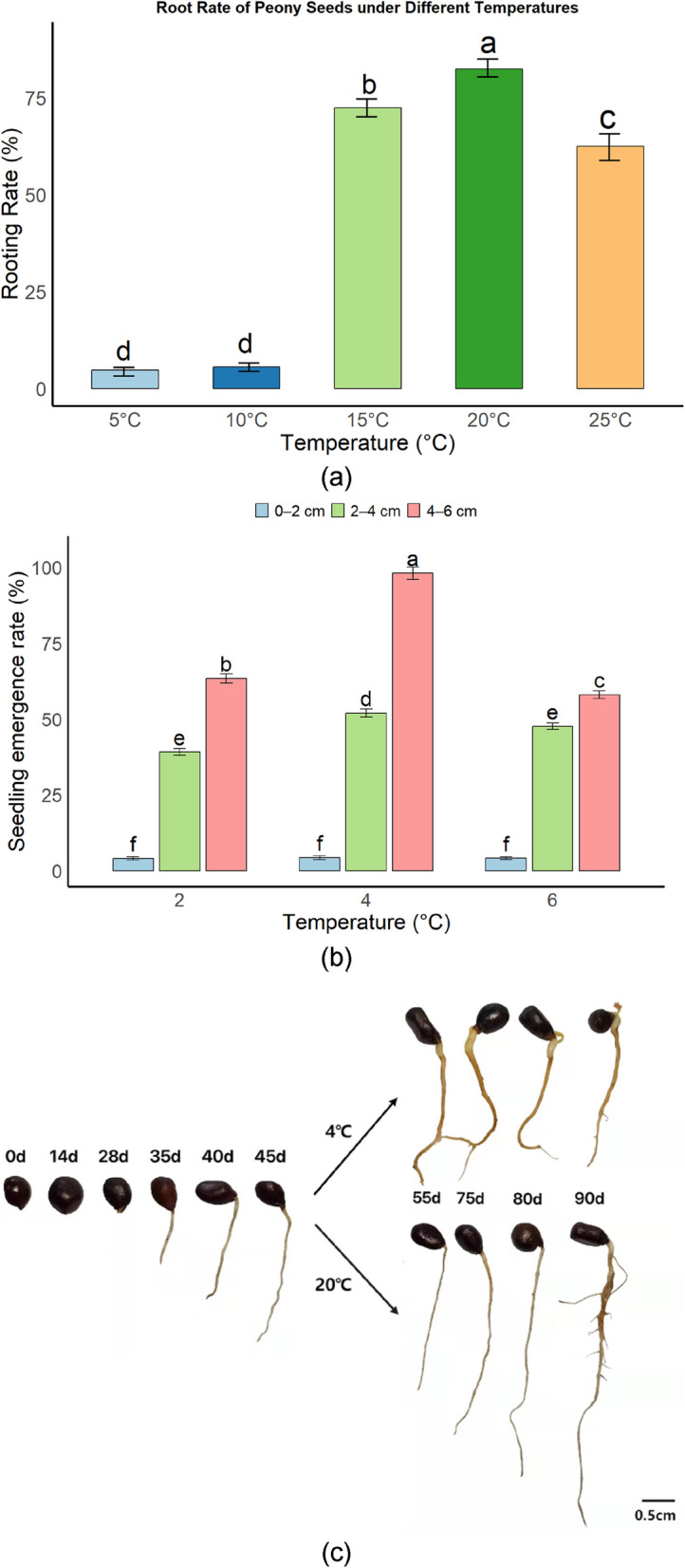
Effect of temperature on rooting and germination in *P. lactiflora* Seeds. **a** Rooting rate of *P. lactiflora* seeds under different warm temperature treatments (5 °C, 10 °C, 15 °C, 20 °C, 25 °C). Different letters indicate significant differences between groups (*P* < 0.05). **b** Germination rate of *P. lactiflora* seeds under different cold temperature treatments (2 °C, 4 °C, 6 °C), showing changes with different embryo root lengths (0–2 cm, 2–4 cm, 4–6 cm). Bars represent different embryo root length intervals, and different letters indicate significant differences. **c** Sequential images showing morphological changes in *P. lactiflora* seeds during the "20 °C root induction – 4 °C bud initiation" treatment. The 20 °C stage induces root growth, while the 4 °C stage triggers bud germination. Scale bar: 0.5 cm

Significant differences in rooting rates were observed under different temperature conditions. Seeds treated at 20 °C exhibited the highest rooting rate (82.45%), which was markedly higher than that at 15 °C (72.45%) and 25 °C (62.55%). In contrast, rooting rates under 5 °C and 10 °C were both below 6%. Within the range of 15–25 °C, rooting capacity first increased and then declined with rising temperature, suggesting that 20 °C is the optimal temperature for initiating radicle growth (Fig. 1a).

Seedling emergence was significantly affected by the interaction between radicle length and cold stratification temperature. Seeds with radicles shorter than 2 cm exhibited extremely low emergence rates (< 5%) under all tested temperatures (2–6 °C), indicating that insufficient radicle development restricts shoot growth. For seeds with radicle lengths of 2–4 cm, emergence rates varied considerably with temperature, with the 4 °C treatment showing the best performance (52%), significantly higher than those at 2 °C and 6 °C. In the 4–6 cm radicle group, seedling emergence under 4 °C reached near saturation (98.12%), significantly exceeding that of other temperature treatments (*P* < 0.05) (Fig. 1b).

Morphological observations revealed that continuous incubation at 20 °C effectively promoted radicle elongation, while subsequent cold treatment at 4 °C rapidly triggered epicotyl growth and seedling emergence (Fig. 1c). These results indicate that sufficient development of the hypocotyl is a prerequisite for epicotyl dormancy release, and a sequential warm–cold stratification regime provides the optimal conditions for *P. lactiflora* seed germination.

### Changes in nutrient content and hydrolytic enzyme activity during dormancy release in *P. lactiflora* seeds

**Fig. 2:**
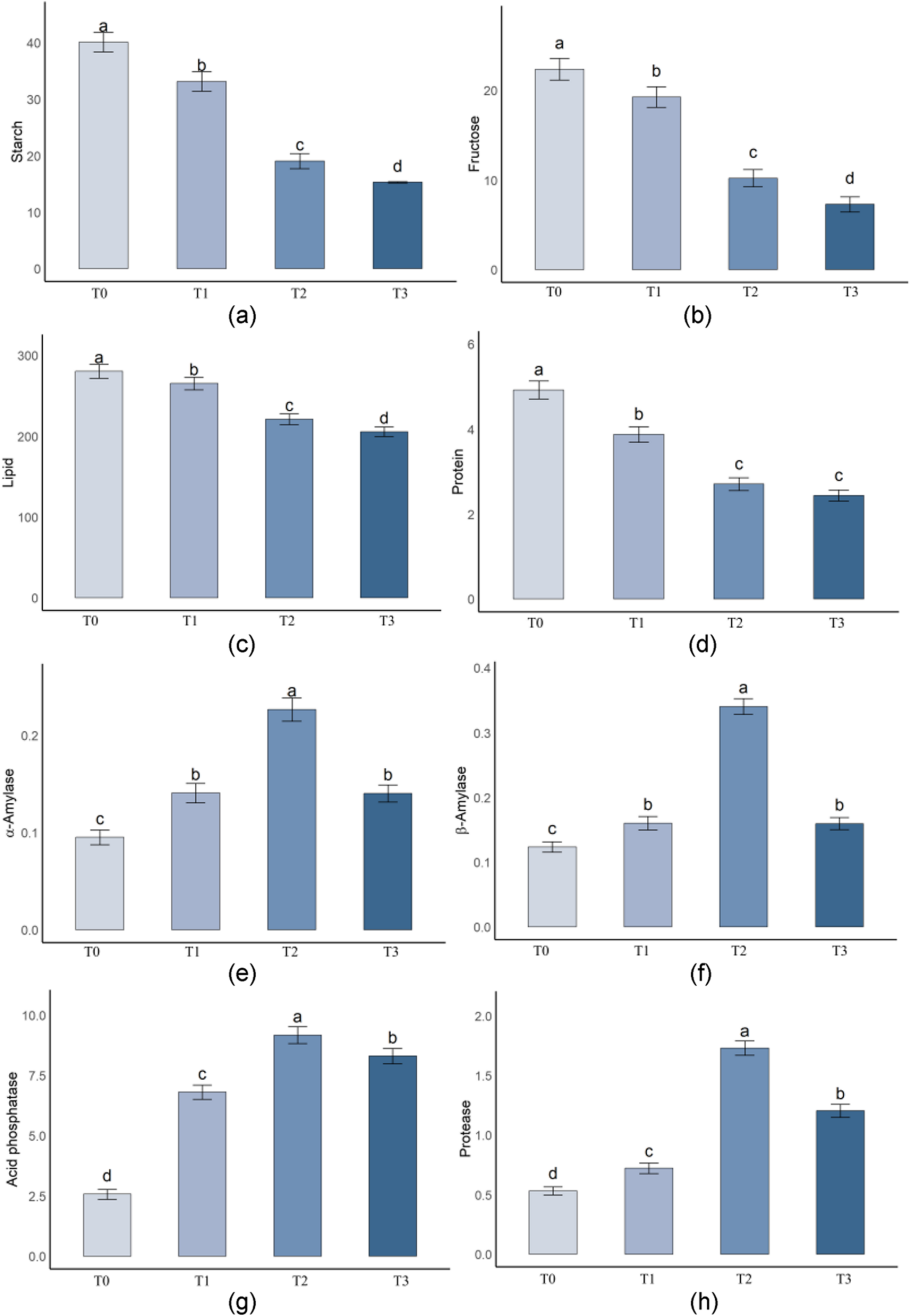
Changes in nutrient content and hydrolytic enzyme activities in *P. lactiflora* seeds during cold stratification. **a** Starch; (**b**) Fructose; (**c**) Lipid; (**d**) Protein; (**e**) α-Amylase; (**f**) β-Amylase; (**g**) Acid phosphatase; (**h**) Protease. Bars represent means ± SD (*n* = 3). Different lowercase letters indicate significant differences among stratification time points (*P* < 0.05)

To elucidate the stage-specific roles of nutrient mobilization and metabolic enzyme activities during dormancy release in *P. lactiflora* seeds, four key time points were selected for dynamic analysis: T0 (0 days), T1 (28 days), T2 (55 days), and T3 (80 days), corresponding to the early and late phases of warm stratification (20 °C for 0–45 days) and the early and late phases of cold stratification (4 °C from day 45 onwards). We examined the contents of starch, fructose, lipids, and proteins, as well as the activities of α-amylase, β-amylase, acid phosphatase, and protease at each stage (Fig. 2a–h).

During the warm stratification phase (T0–T1), which primarily induced hypocotyl (radicle) elongation, only moderate decreases were observed in nutrient contents, and the activities of hydrolytic enzymes remained at low levels. This suggests that although root growth was initiated, large-scale metabolic mobilization had not yet commenced.

In contrast, the cold stratification phase (T1–T3) triggered a significant physiological transition as epicotyl dormancy was progressively released and seedling emergence began. This phase was marked by a rapid mobilization of storage compounds and strong induction of key hydrolytic enzymes.

Starch content significantly decreased over time (*P* < 0.05), from 40.13% at T0 to 15.34% at T3 (Fig. 2a), indicating that carbohydrate reserves were extensively degraded to meet energy demands. Similarly, fructose content declined from 22.34% to 7.31% (Fig. 2b), likely due to its utilization in supporting epicotyl growth. Lipid and protein contents also decreased markedly (Fig. 2c, d), with lipid content dropping from 280.13 mg/g to 205.76 mg/g and protein content from 4.92% to 2.44%, suggesting that both serve as important energy and structural sources during germination.

The activities of hydrolytic enzymes were significantly enhanced during cold stratification. α-Amylase and β-amylase activities increased sharply and peaked at T2 (0.2267 U/mg and 0.3410 U/mg, respectively), then slightly declined (Fig. 2e, f), highlighting their key roles in starch degradation and sugar release. Acid phosphatase activity continuously increased and reached its maximum at T2 (9.17 U/mg) (Fig. 2g), suggesting its involvement in organic phosphorus hydrolysis and phosphorus supply to embryonic tissues. Protease activity also peaked at T2 (1.73 U/mg), and although it slightly decreased at T3, it remained significantly higher than at T0 (Fig. 2h), reflecting active degradation of storage proteins during this stage.

In summary, *P. lactiflora* seeds exhibited clear stage-specific nutritional and metabolic reprogramming under a sequential warm–cold stratification regime. Warm stratification at 20 °C primarily facilitated radicle emergence, whereas subsequent cold stratification at 4 °C strongly activated nutrient degradation and enzyme activity, creating favorable metabolic conditions for epicotyl elongation and seedling emergence. Notably, T2 (early cold stratification) emerged as a critical transition point where nutrient breakdown and enzymatic activation synergistically peaked, marking the shift from dormancy maintenance to germination initiation.

### Changes in endogenous hormone levels during dormancy release in *P. lactiflora* seeds

**Fig. 3:**
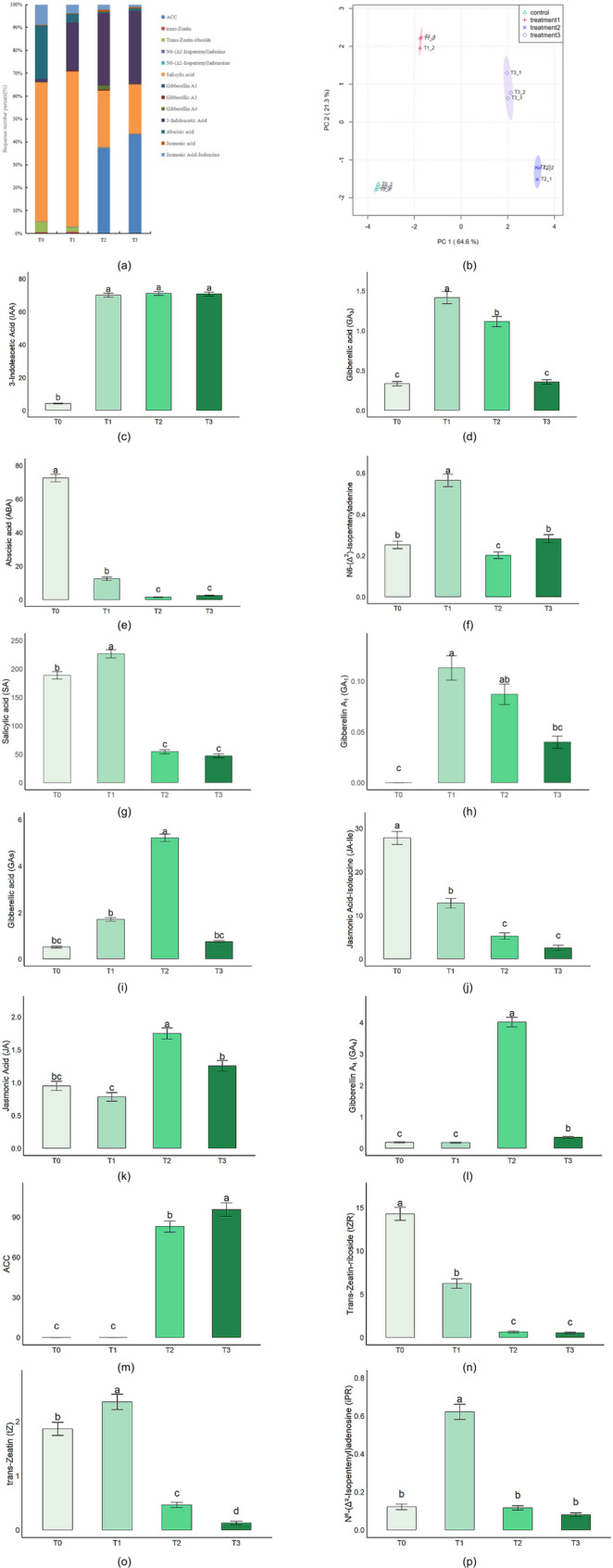
Dynamic changes in 14 endogenous phytohormones in *P. lactiflora* seeds during cold stratification. **a** Proportional composition of 14 phytohormones at four stratification stages (T0, T1, T2, T3); (**b**) Principal component analysis (PCA) based on phytohormone contents across all samples; (**c**) Indole-3-acetic acid (IAA); (**d**) Gibberellin A₃ (GA₃); (**e**) Abscisic acid (ABA); (**f**) N⁶-(Δ^2^-Isopentenyl)adenine (N6-iP); (**g**) Salicylic acid (SA); (**h**) Gibberellin A₁ (GA₁); (**i**) Total gibberellins (GAs); (**j**) Jasmonic acid-isoleucine (JA-Ile); (**k**) Jasmonic acid (JA); (**l**) Gibberellin A₄ (GA₄); (**m**) 1-Aminocyclopropane-1-carboxylic acid (ACC); (**n**) trans-Zeatin riboside (tZR); (**o**) trans-Zeatin (tZ); (**p**) N⁶-(Δ^2^-Isopentenyl)adenosine (iPR). Bar plots show the mean ± SD (*n* = 3) at four stratification stages (T0, T1, T2, T3). Different lowercase letters indicate significant differences among time poin (*P* < 0.05). All values are expressed in ng·g⁻^1^ fresh weight (FW)

To comprehensively reveal the dynamic characteristics of hormone regulation during the cold stratification process in *P. lactiflora* seeds, we first visualized the relative proportions of 14 major plant hormones (Fig. 3a) and further explored the differences between samples at different treatment stages using Principal Component Analysis (PCA) (Fig. 3b). The results showed significant differences in hormone composition at different cold stratification stages: during the warm stratification phase (0–45 days, 20 °C), T0 was dominated by abscisic acid (ABA), indicating its core role in dormancy maintenance; in T1, auxin (IAA) and gibberellins (GA₃) became the dominant hormones, accounting for over 70%, suggesting that this phase is primarily focused on promoting growth; during the cold stratification phase (45–80 days, 4 °C), T2 exhibited a more complex hormonal profile with high levels of IAA and GA, while stress-related hormones such as jasmonic acid (JA), salicylic acid (SA), and ACC increased, reflecting a more complex signaling regulation in the later stages; by T3, ACC was the highest among the hormones, highlighting the importance of ethylene precursor synthesis in the embryo axis breaking through the seed coat. PCA further confirmed the significant differences in hormone profiles between the stages. The first principal component (PC1) explained 64.6% of the total variance, and the second principal component (PC2) explained 21.3%. Samples at different treatment time points formed distinct clusters in the PCA space, with the greatest difference between T0 and T1, and T2 and T3 clustering closely together, indicating good reproducibility within groups and confirming the stability and reliability of the hormone expression data. The hormonal regulation in cold stratification clearly exhibits stage-specific differentiation.

To investigate the temporal regulation of plant hormones in dormancy release, we further examined the changes in 14 endogenous hormones at four cold stratification periods (T0: 0 days, T1: 28 days, T2: 55 days, T3: 80 days).

IAA levels significantly increased at T1 (70.09 ng/g), nearly 17 times higher than at T0 (4.32 ng/g) (*P* < 0.05), and remained high at T2 and T3 (Fig. 3c). This indicates that IAA synthesis is rapidly activated early in cold stratification, potentially regulating cell division and differentiation during the early stages of dormancy release. Gibberellins GA₃ and GA₁ also increased significantly at T1 and T2, with GA₃ reaching its peak at T1 (1.41 ng/g) (Fig. 3d), and GA₁ and GA₄ reaching 0.087 ng/g and 4.015 ng/g, respectively, at T2 (Fig. 3h, l). Additionally, total gibberellins (GAs) reached a peak at T2 (5.22 ng/g), suggesting that GA hormones are actively synthesized during the middle phase of cold stratification, promoting embryo elongation and seed germination (Fig. 3i).

In contrast, ABA levels continuously decreased throughout cold stratification, from 72.54 ng/g at T0 to 1.49 ng/g at T2, and remained low at T3, indicating that the inhibitory hormone ABA is rapidly degraded, which is one of the key mechanisms for breaking dormancy (Fig. 3e). Cytokinin levels exhibited dynamic changes. N6-(Δ^2^-isopentenyl) adenosine (N6-iP) significantly increased at T1, and its adenosine form (iPR) also rose to 0.621 ng/g at T1, followed by a decline (Fig. 3f, p). The levels of tZR and tZ were highest at T0 and significantly decreased afterward, suggesting that different forms of cytokinins may play stage-specific roles in dormancy release (Fig. 3n, o).

In the jasmonic acid pathway, JA-Ile content steadily decreased during cold stratification, from 27.79 ng/g at T0 to 2.56 ng/g at T3, while JA peaked at T2 (1.75 ng/g), potentially related to stress responses and late-stage germination regulation (Fig. 3j, k). Ethylene precursor ACC was almost undetectable at T0 and T1 but increased significantly at T2 (82.95 ng/g) and reached 95.64 ng/g at T3, indicating that ethylene synthesis is activated in the later stages, possibly facilitating the breaking of the seed coat by the epicotyl (Fig. 3m). Salicylic acid (SA) levels significantly increased at T1 (226.35 ng/g), then sharply declined, possibly playing a role in defense or redox regulation during early dormancy release (Fig. 3g).

In conclusion, *P. lactiflora* seeds exhibited typical characteristics during cold stratification, with ABA rapidly declining, IAA and GA hormones increasing, dynamic regulation of cytokinins, and late-stage activation of ethylene and JA. These results reveal that dormancy release is a complex process of multi-hormonal synergistic regulation and stage-specific hormonal remodeling. Notably, T1 (28 days) was identified as the key period with the most pronounced hormonal changes, laying the physiological foundation for embryo axis breakthrough and dormancy release.

### Transcriptome analysis

#### Transcriptome sequencing quality and differentially expressed gene (DEG) statistics

To systematically reveal the transcriptional regulatory mechanisms during dormancy release and germination in *P. lactiflora* seeds, we conducted transcriptome sequencing on 12 samples. A total of 83.82 G of high-quality clean data was obtained, with the effective data per sample ranging from 6.75 to 7.25 G. The Q30 value for all samples was no less than 92.49%, and the average GC content was 45.69%, indicating that the sequencing quality was satisfactory and providing a reliable basis for subsequent analyses (Table 1).

**Table 1:** Statistics of sequencing data of peony

Sample	Clean reads	Clean bases	GC Content	% ≥ Q30
T0-1	47.57 M	6.80G	46.42%	94.68%
T0-2	49.47 M	7.06G	46.44%	94.50%
T0-3	50.78 M	7.25G	46.36%	95.01%
T1-1	49.49 M	7.07G	46.78%	94.06%
T1-2	48.60 M	6.94G	46.85%	94.32%
T1-3	50.63 M	7.23G	46.76%	94.57%
T2-1	47.17 M	6.76G	44.86%	94.21%
T2-2	49.50 M	7.13G	44.81%	92.49%
T2-3	48.56 M	7.09G	44.70%	94.75%
T3-1	47.77 M	6.83G	44.55%	94.35%
T3-2	48.25 M	6.91G	44.73%	95.34%
T3-3	47.37 M	6.75G	45.03%	94.43%

**Fig. 4:**
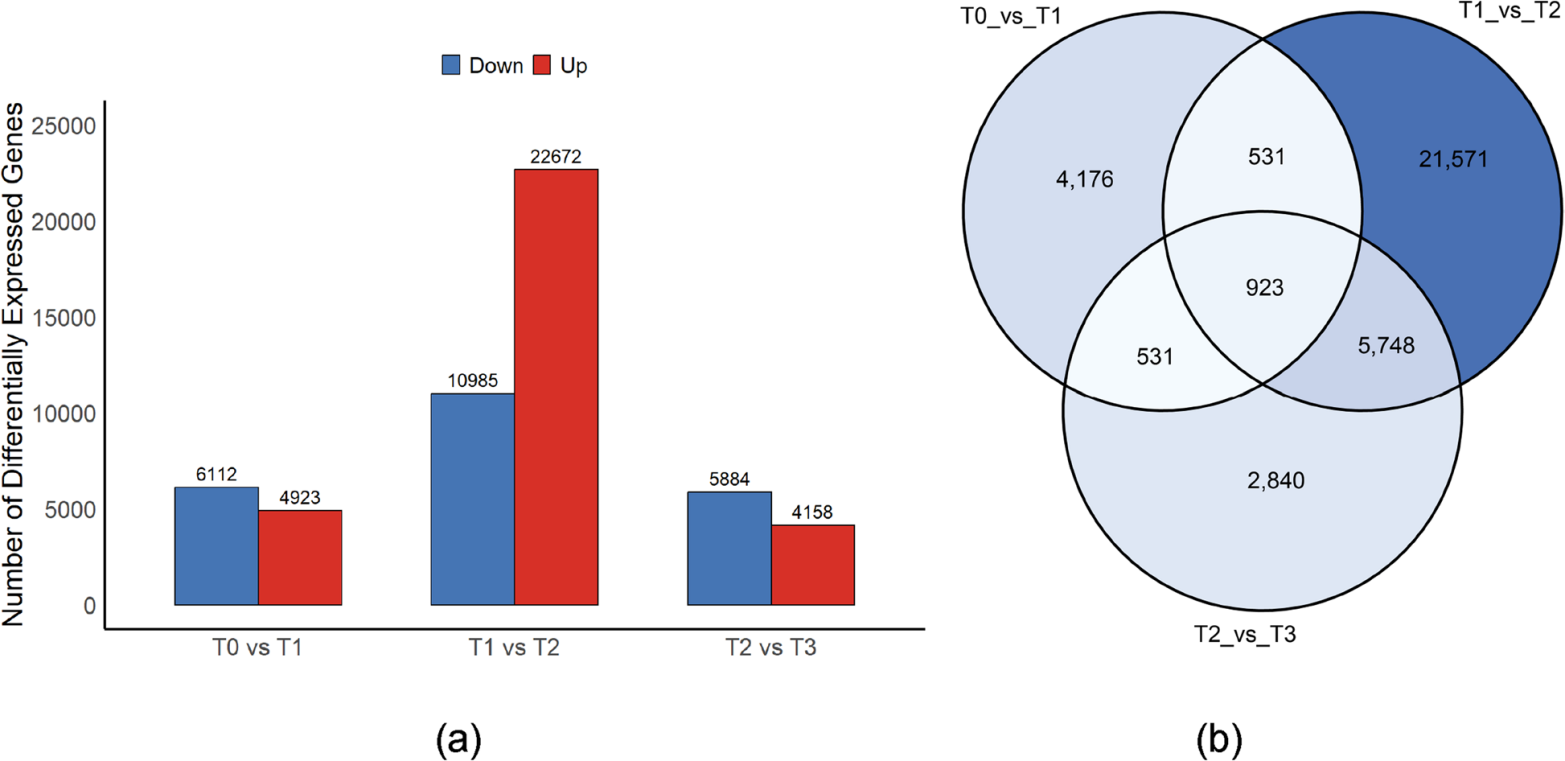
Differentially expressed genes (DEGs) in *P. lactiflora* seeds at different cold stratification stages. **a** The number of upregulated and downregulated DEGs in the comparison groups (T0 vs T1, T1 vs T2, T2 vs T3). **b** Venn diagram of DEGs from the three comparison groups

Based on these data, differentially expressed genes (DEGs) were identified using the criteria of FoldChange ≥ 2 and q-value < 0.05. The results revealed significant transcriptomic differences in *P. lactiflora* seeds at various developmental stages. During the hypocotyl elongation stage, a total of 11,045 DEGs were detected, of which 4,933 genes were upregulated, and 6,112 genes were downregulated. In the epicotyl elongation and cotyledon development stages, 10,042 DEGs were identified, with 4,158 genes upregulated and 5,884 genes downregulated (Fig. 4a). Further analysis showed that 923 DEGs were common across all three stages, suggesting that these genes with consistent expression changes may play a crucial role in breaking seed dormancy (Fig. 4b).

#### Differential gene expression trend analysis and enriched pathways

**Fig. 5:**
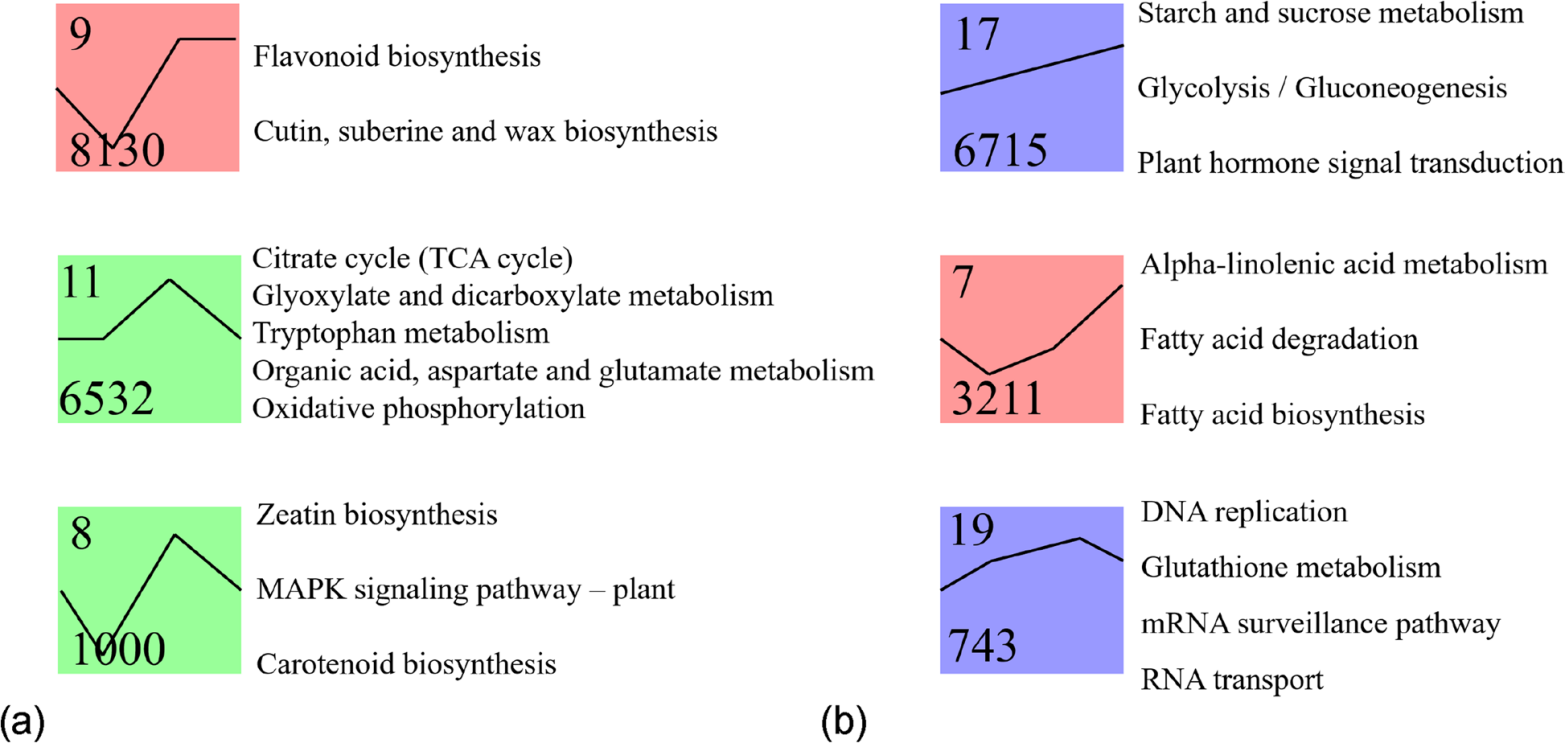
Differentially expressed genes (DEGs) in *P. lactiflora* seeds at different cold stratification stages. **a** The number of upregulated and downregulated DEGs in the comparison groups (T0 vs T1, T1 vs T2, T2 vs T3). **b** Venn diagram of DEGs from the three comparison groups

To further reveal the dynamic changes in gene expression in *P. lactiflora* seeds under cold stratification, a trend clustering analysis was conducted on the differentially expressed genes (DEGs). A total of 20 expression trend modules were identified, with 6 modules showing statistically significant enrichment in their expression patterns (*P* < 0.05) (Fig. 5). The pathways enriched in these significant modules primarily involve metabolic regulation, signal transduction, and genetic information processing, reflecting the multi-level regulatory mechanisms during seed dormancy release.

Module 9 was the largest module, containing 8,130 DEGs, and its expression pattern showed a continuous upward trend. This module was significantly enriched in the biosynthesis pathways of phenylpropanoid compounds and the synthesis of the cuticle, suberin, and wax, suggesting that these structural metabolic activities are significantly enhanced as the seeds gradually activate. Module 17, the second largest module (6,715 DEGs), was mainly involved in starch and sucrose metabolism, glycolysis/gluconeogenesis, and plant hormone signal transduction pathways. Its overall expression level continuously increased, indicating that energy metabolism and hormone regulation play key roles during the embryo axis elongation and cotyledon activation stages.

Module 11, containing 6,532 DEGs, was enriched in several basic metabolic pathways, including the TCA cycle, organic acid metabolism, tryptophan and glutamate metabolism, and oxidative phosphorylation, suggesting that energy and amino acid metabolism are highly active during later-stage embryo tissue development. Module 7, which includes 3,211 genes, was significantly enriched in lipid metabolism pathways, such as fatty acid biosynthesis, fatty acid degradation, and α-linolenic acid metabolism, suggesting that lipid metabolism may provide an energy source for seed germination and participate in the generation of signaling molecules.

Furthermore, Module 8 (1,080 DEGs) was mainly enriched in the biosynthesis of zeatin, carotenoid synthesis, and MAPK signaling pathways, indicating that cell signaling and hormone regulation work synergistically during the different stages of seed development. Module 19 (743 DEGs) was enriched in genetic information processing and antioxidant-related pathways, including DNA replication, RNA transport, mRNA surveillance, and glutathione metabolism. This suggests that the gene expression regulation system and cellular protection mechanisms are also of great importance during seed germination.

In conclusion, the trend analysis of differentially expressed genes reveals the metabolic reprogramming and signal regulation mechanisms during dormancy release and germination in *P. lactiflora* seeds. This provides a crucial foundation for further elucidating the key regulatory factors involved in seed development.

### WGCNA analysis

#### Identification of key modules related to dormancy release in *P. lactiflora* seeds

To systematically identify functional modules that are significantly correlated with physiological and hormonal indicators during seed dormancy release in *P. lactiflora*, we performed weighted gene co-expression network analysis (WGCNA) based on 22 traits, including starch, fructose, protein, lipid content, the activity of various hydrolytic enzymes (α-amylase, β-amylase, acid phosphatase, protease), and the concentrations of 14 plant hormones.

**Fig. 6:**
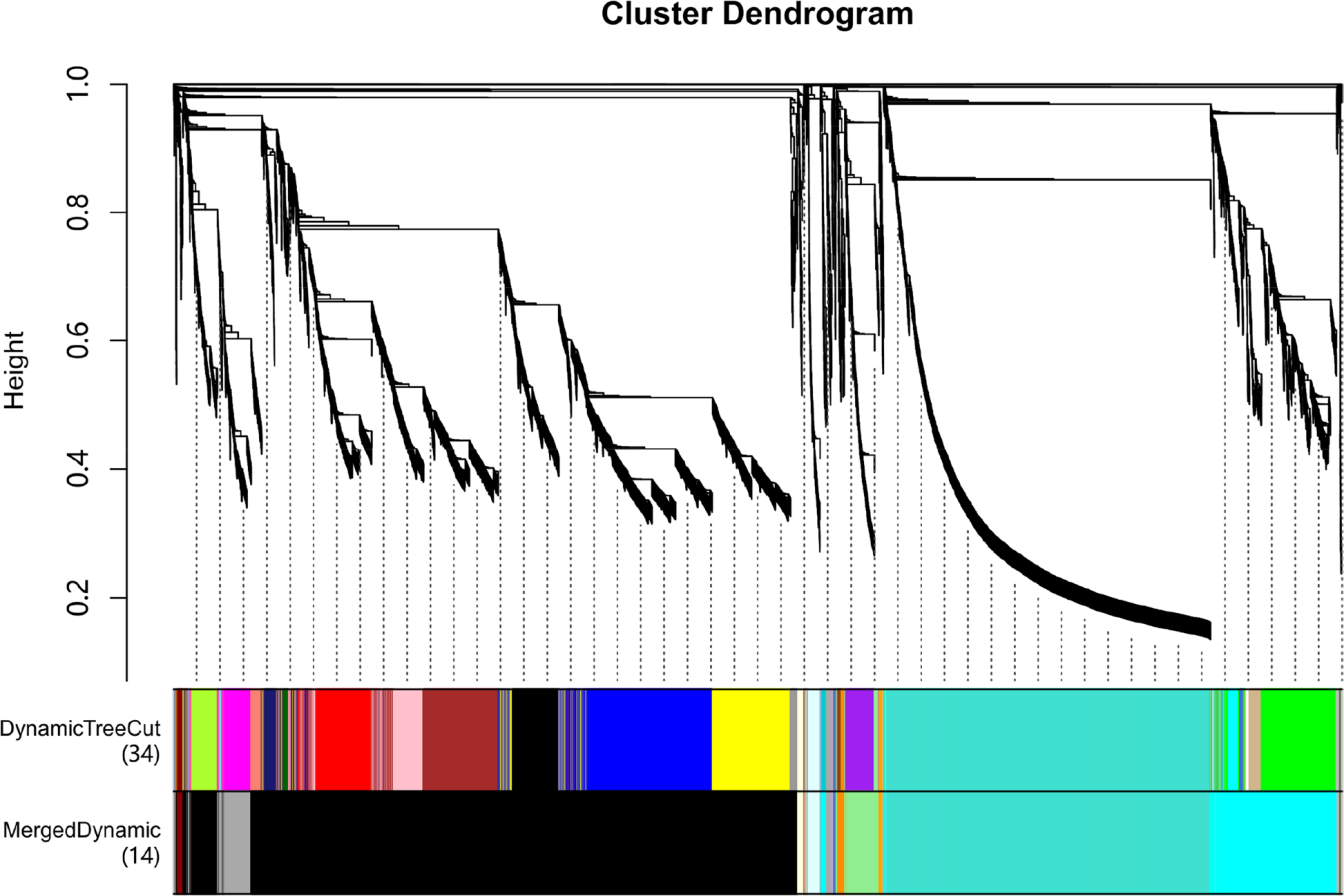
WGCNA module identification for key traits in *P. lactiflora* seed dormancy release

The preliminary gene clustering tree (Cluster Dendrogram) is shown in the figure. WGCNA identified 34 initial modules (DynamicTreeCut), which were subsequently merged into 14 co-expression modules (MergedDynamic) using a similarity-based merging strategy. Each module, represented by a distinct color, contains a set of genes with highly correlated expression patterns (Fig. 6).

There was significant expression heterogeneity between modules, reflecting the differential response of multiple gene sets to changes in hormone levels and nutrient metabolism dynamics at different stages of cold stratification in *P. lactiflora* seeds. Notably, larger and more clearly differentiated modules, such as the blue, yellow, cyan, and black modules, were identified in the clustering diagram, suggesting their potential biological significance.

#### WGCNA reveals the modules and potential functional relationships associated with key traits of dormancy release in *P. lactiflora* seeds

**Fig. 7:**
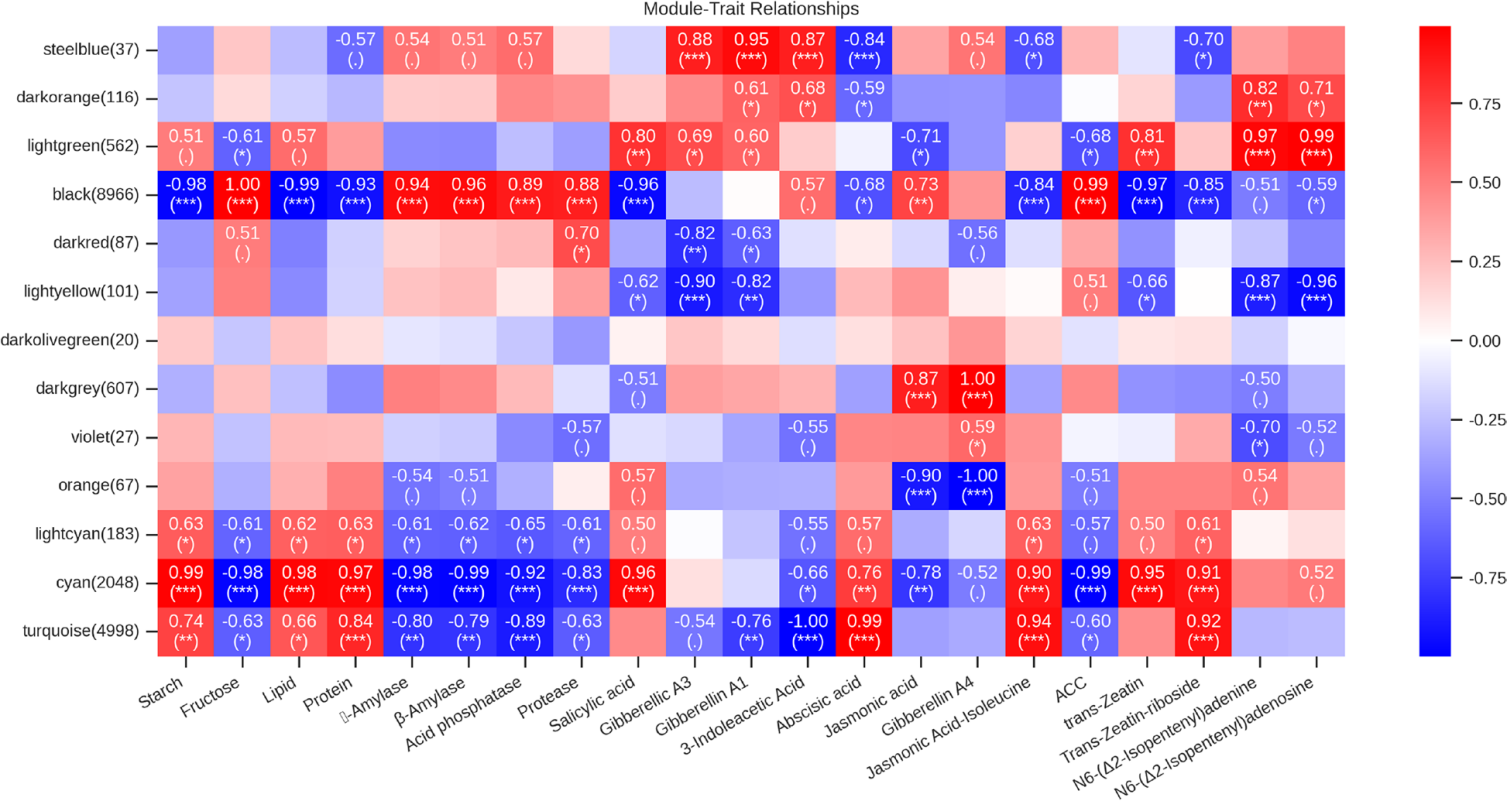
Module-trait correlation heatmap. Each cell represents the Pearson correlation coefficient between the module eigengene and the corresponding phenotype traits. The color scale ranges from red (positive correlation) to blue (negative correlation), indicating the strength of the correlation. The significance levels are indicated in parentheses (*P < 0.05, **P < 0.01, ***P < 0.001)

To further clarify the relationships between co-expression modules and major physiological, biochemical, and hormonal indicators during the dormancy release process in *P. lactiflora* seeds, we constructed a module-trait correlation heatmap (Fig. 7) based on the WGCNA analysis results. This heatmap encompasses 22 phenotypic traits and 14 gene expression modules. The correlation coefficients and significance (p-value) were used to screen for key modules, identifying regulatory units closely associated with specific physiological processes.

Among all the modules, the black module (containing 8,966 genes) exhibited the most widespread and significant negative correlations with nearly all physiological indicators (Starch, Protein, α/β-Amylase, Acid Phosphatase, Protease) and hormonal indicators (GA₃, GA₁, IAA, ACC, etc.), with a strong negative correlation (|r|≥ 0.9, *P* < 0.001). This suggests that the black module may be enriched in negative regulatory factors involved in dormancy maintenance or inhibition of germination.

In contrast, the cyan module (containing 2,048 genes) showed significant positive correlations with most hormones (GA₃, GA₁, IAA, JA, SA) and hydrolytic enzyme activity indicators (α/β-Amylase, Protease), with correlation coefficients generally above 0.9 (*P* < 0.001). This indicates that the cyan module may regulate nutrient mobilization and hormone response, working synergistically to promote the transition of seeds from dormancy to germination.

The turquoise module (containing 4,998 genes) was also significantly positively correlated with hormone indicators (GA₃, GA₁, IAA, JA) and showed positive correlations with protein and lipid metabolism traits (e.g., with Protein: *r* = 0.66, *P* < 0.01; Lipid: *r* = 0.66, *P* < 0.05). This suggests that the turquoise module may be involved in energy metabolism and cell activation. Additionally, the light cyan module warrants attention as it showed significant positive correlations with hormones such as GA₃, IAA, and JA, but exhibited highly negative correlations with protease (*r* = −0.83) and acid phosphatase (*r* = −0.99) (*P* < 0.001), potentially representing a relatively independent regulatory pathway.

In summary, the black, cyan, and turquoise modules were identified as key functional modules closely related to hormone metabolism and energy mobilization during the dormancy release process in *P. lactiflora* seeds. Based on these findings, further pathway enrichment analysis and core hub gene screening will be conducted to further elucidate their regulatory roles in the transition from seed dormancy to germination.

#### Co-expression network characteristics and hub gene identification of key modules

In the WGCNA co-expression network constructed in this study, the black, cyan, and turquoise modules were identified as key modules significantly associated with the dormancy release process in *P. lactiflora* seeds. These modules exhibited distinct features in terms of network structure, expression dynamics, and functional associations. By integrating the identification of hub genes (key genes), co-expression network diagrams, and gene expression heatmaps, we systematically revealed the potential regulatory roles of these modules during the different stages of dormancy release.

**Fig. 8:**
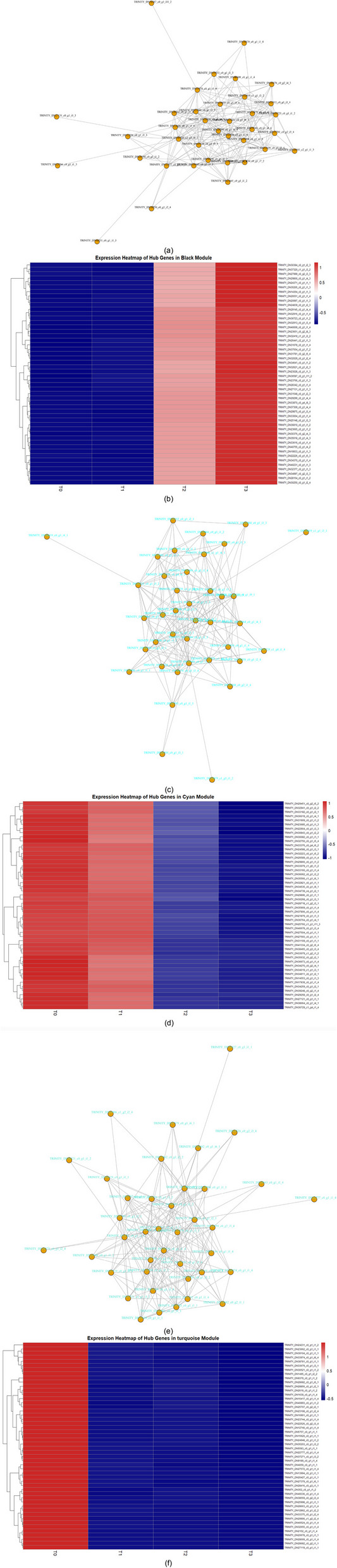
Co-expression network and expression pattern analysis of key genes in different modules. **a** Co-expression network of the Black module. **b** Standardized expression heatmap of key genes in the Black module. **c** Co-expression network of the Cyan module. **d** Expression heatmap of key genes in the Cyan module. **e** Co-expression network of the Turquoise module. **f** Expression heatmap of key genes in the Turquoise module

The black module (Fig. 8a, b) exhibited a highly compact network structure, with dense gene connections and efficient information transfer. The expression heatmap showed that the hub genes in this module had very low expression levels during T0 and T1, but they significantly increased at T2 and peaked at T3, reflecting a typical “late activation” expression pattern. This trend aligns closely with the physiological transition of seeds from dormancy to germination. The highly connected hub genes in this module, such as *TRINITY_DN40755_c2_g1_i4_2*, *TRINITY_DN31548_c0_g1_i2_3*, and *TRINITY_DN23651_c0_g2_i8_3*, were significantly associated with starch degradation, gibberellin metabolism, and IAA response, suggesting their core role in regulating embryo axis elongation, cell wall loosening, and hormone balance. In the module-trait correlation heatmap, this module was significantly positively correlated with germination-related factors such as α-amylase, GA₃, and IAA (*P* < 0.001), further emphasizing its functional role in embryo activation and metabolic activation.

The cyan module (Fig. 8c, d) also exhibited a clear network topology, but its expression heatmap revealed a significant “high expression at early stages, followed by rapid downregulation” pattern. The expression level of this module was generally higher at T0 and T1, but quickly suppressed at T2 and T3, suggesting its potential key role in dormancy maintenance or cold stress response. Hub genes in the network, such as *TRINITY_DN34535_c0_g1_i9_1*, *TRINITY_DN32941_c0_g1_i2_2*, and *TRINITY_DN35203_c0_g1_i3_2*, formed strong correlation centers and may encode functional elements related to ethylene biosynthesis, ABA signaling negative regulation, or cold-induced proteins. This module was positively correlated with ABA, JA, and antioxidant enzyme activity, but negatively correlated with GA and embryo axis elongation traits, supporting its possible role in transcriptional regulation mechanisms during the early cold stratification phase, suppressing embryo activation and maintaining dormancy.

The turquoise module (Fig. 8e, f), although relatively looser in network topology, exhibited a highly characteristic expression pattern. Most of the hub genes in this module showed significantly higher expression at T0 compared to other stages, followed by a continuous downregulation, with nearly complete silencing at T3. This suggests a strong activation during the early dormancy maintenance phase. Key hub genes such as *TRINITY_DN24321_c0_g1_i1_2*, *TRINITY_DN23662_c0_g1_i1_3*, and *TRINITY_DN21904_c0_g1_i1_2* may be involved in signal transduction regulation, maintaining redox balance, or regulating storage substance synthesis. This module was positively correlated with ABA content and the accumulation of tZR and other cytokinin forms, indicating its role in the cold-induced regulation of defense-related pathways and maintaining seed dormancy, thus creating a molecular barrier in preparation for germination.

In summary, the three key modules represent different stages and functional roles during the dormancy release process in *P. lactiflora* seeds: the black module represents the core network for metabolic activation and embryo axis initiation, the cyan module drives early-stage suppression and cold response pathways, and the turquoise module forms an early hormone response and transcriptional homeostasis regulation system. Within each module, hub genes have high connectivity and expression regulation strength, potentially acting as “master switches” in controlling transcriptional programs. This study, by integrating co-expression network structures, expression patterns, and hub gene identification, provides crucial insights and candidate targets for further elucidating the molecular basis of seed dormancy release.

### Seed dormancy release and germination-related plant hormone metabolism gene expression patterns

#### Expression pattern analysis of abscisic acid (ABA) synthesis and signal transduction-related genes

**Fig. 9:**
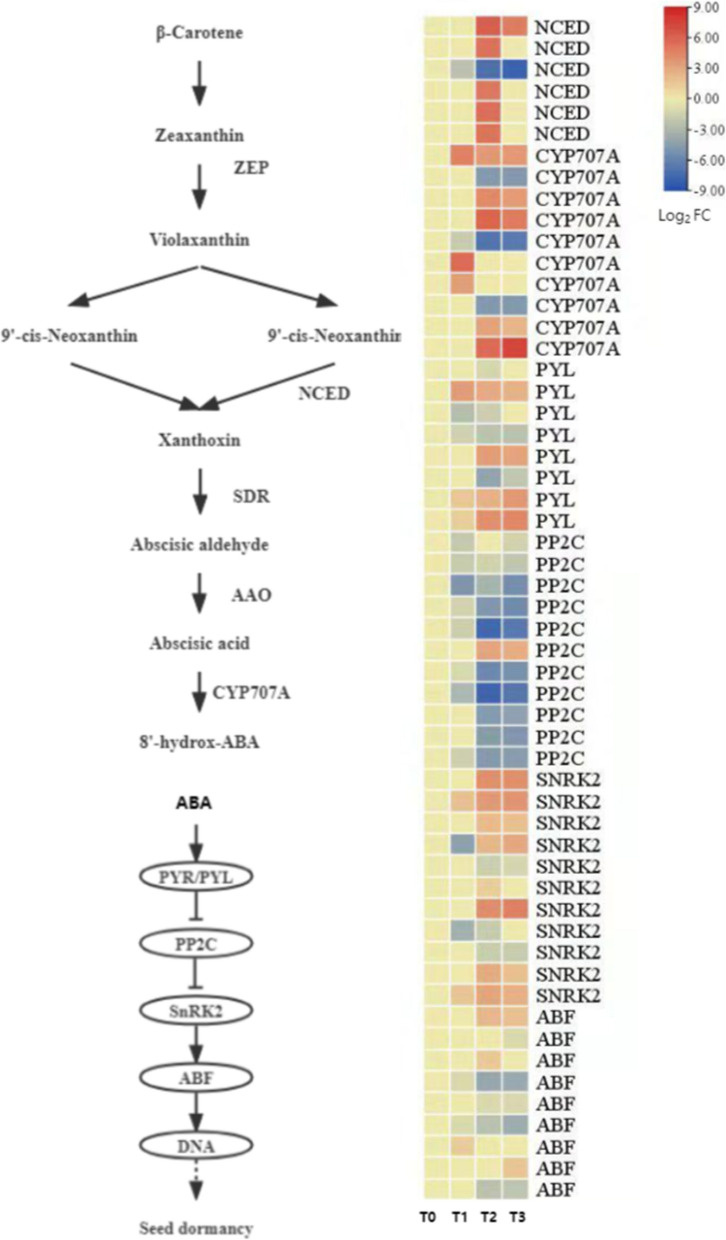
ABA biosynthesis and signaling pathway in* P. lactiflora* Seeds During Dormancy Release. The diagram illustrates the key genes involved in ABA biosynthesis and signal transduction, along with their expression patterns at different cold stratification stages (T0, T1, T2, T3). The heatmap on the right shows the Log2 fold change (Log2 FC) values of differential gene expression (DEGs) for each key gene

Abscisic acid (ABA) plays a central role in regulating seed dormancy establishment and release. This study systematically analyzed the expression changes of ABA synthesis, metabolism, and signal transduction-related genes during the cold stratification-induced dormancy release process in *P. lactiflora* seeds, constructing its regulatory mechanism map (Fig. 9).

ABA synthesis mainly relies on the carotenoid pathway, where β-carotene is catalyzed by a series of enzymes to generate ABA precursors. In *P. lactiflora* seeds, six differentially expressed genes (DEGs) encoding 9-cis-epoxycarotenoid dioxygenase (NCED), a key enzyme in ABA synthesis, were successfully identified. Of these, five DEGs were significantly upregulated from T0 to T3, suggesting that ABA synthesis is active during the early stages of dormancy. In contrast, one *NCED* family member showed significant downregulation with the greatest fold change, possibly indicating a specific regulatory role.

Regarding ABA metabolism, ten DEGs encoding ABA 8'-hydroxylase (CYP707A), responsible for ABA catabolism, were detected. Most of these genes (7 DEGs) showed increased expression from T1 to T3, which was consistent with the downward trend in ABA content, indicating that *P. lactiflora* seeds enhance metabolism to reduce ABA accumulation during dormancy release.

At the signal transduction level, this study identified eight ABA receptor genes (*PYL*), eleven protein phosphatase 2 C (*PP2C*) genes, and eleven SnRK2-like kinase DEGs. During dormancy release, ABA binds to the *PYR/PYL* complex, inhibiting *PP2C* activity, which releases its inhibition of *SnRK2* and activates its phosphorylation activity. This process then regulates the downstream ABA-responsive element-binding factors (*ABFs*), ultimately triggering the expression of genes related to seed germination. The data show that, during the T2 to T3 stages, the majority of *PP2C* genes were significantly downregulated, while the expression of nine *SnRK2* members was significantly upregulated, supporting the activation of the ABA signaling pathway during dormancy release.

In conclusion, the ABA signaling pathway in *P. lactiflora* seeds during dormancy release follows a typical pattern of "early synthesis enhancement, later metabolism acceleration, and signal transduction activation," suggesting that ABA, by regulating synthesis, metabolism, and signaling responses, plays a pivotal role in breaking the dormancy state.

#### Expression pattern analysis of gibberellin (GA) synthesis and signal transduction-related genes

**Fig. 10:**
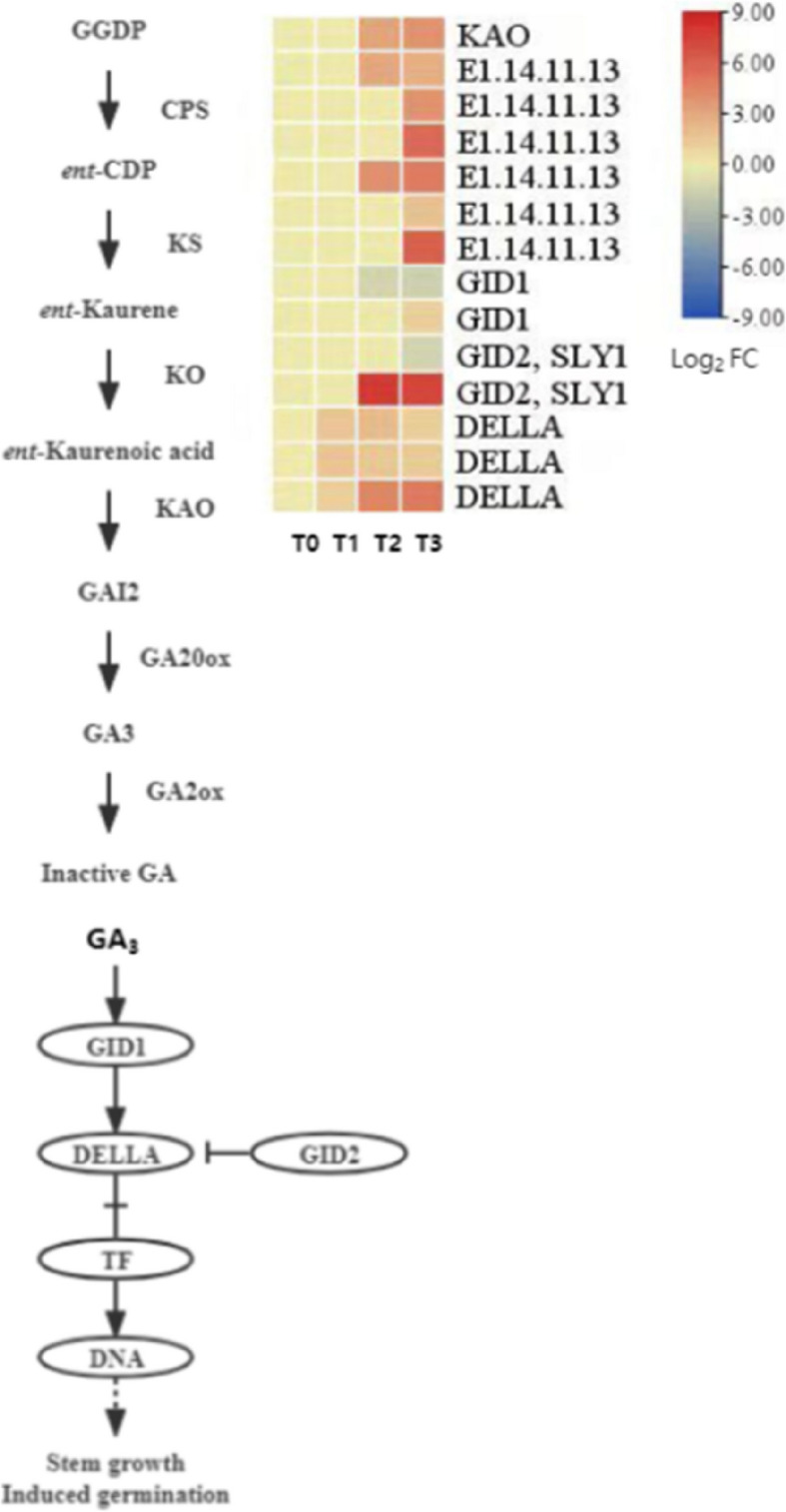
GA Biosynthesis and Signaling Pathway in *P. lactiflora* Seeds During Dormancy Release. This diagram illustrates the key genes involved in GA biosynthesis and signal transduction pathways, along with their expression patterns at different cold stratification stages (T0, T1, T2, T3). The heatmap on the right shows the Log2 fold change (Log2 FC) values of differential gene expression (DEGs) for each key gene

Gibberellin (GA) is one of the key hormones promoting seed germination and plays an important role in the dormancy release process of *P. lactiflora* seeds. This study systematically analyzed the expression characteristics of GA synthesis, metabolism, and signal transduction-related genes, as shown in Fig. 10.

The GA biosynthesis pathway can be divided into three main stages: first, the synthesis of ent-kaurene as an intermediate through enzymatic reactions, followed by multi-step oxidation to form GA₁₂-aldehyde, and finally, the formation of bioactive GAs such as GA₁ and GA₄ through the catalytic actions of GA20 oxidase (*GA20ox*) and GA3 oxidase (*GA3ox*). In this study, one DEG encoding ent-kaurene oxidase (*KAO*) was identified, which showed significant upregulation from T0 to T3, indicating enhanced GA biosynthesis activity, consistent with the increase in endogenous GA levels.

Regarding GA inactivation metabolism, six DEGs encoding gibberellin 2-oxidase (*GA2ox*) were identified, which were significantly downregulated at T3. This suggests that the degradation rate of GA decreases in the later stages of germination, favoring the sustained activity of bioactive GAs. However, the final GA content still showed a downward trend, possibly related to feedback regulatory mechanisms.

In the GA signal transduction pathway, GA binds to its receptor *GID1*, leading to the association and ubiquitination degradation of DELLA proteins with *GID2* (or *SLY1*), thus releasing the transcription factor (TF) activity, which activates the expression of downstream germination-related genes. In this study, four signal response-related protein DEGs were identified (including *GID1* and *GID2*), of which one *SLY1/GID2* member was significantly upregulated from T2 to T3, while the expression of three *DELLA* protein DEGs remained relatively low, aligning with the trend of DELLA inhibition being relieved during dormancy release.

In conclusion, during the dormancy release process of *P. lactiflora* seeds, GA synthesis is active, metabolism is downregulated, and the signal transduction pathway is successfully activated, which together drive the transition of seeds from dormancy to germination. These findings provide molecular evidence for the key role of GA in regulating seed germination.

#### Expression pattern analysis of indole-3-acetic acid (IAA) synthesis and signal transduction-related genes

**Fig. 11:**
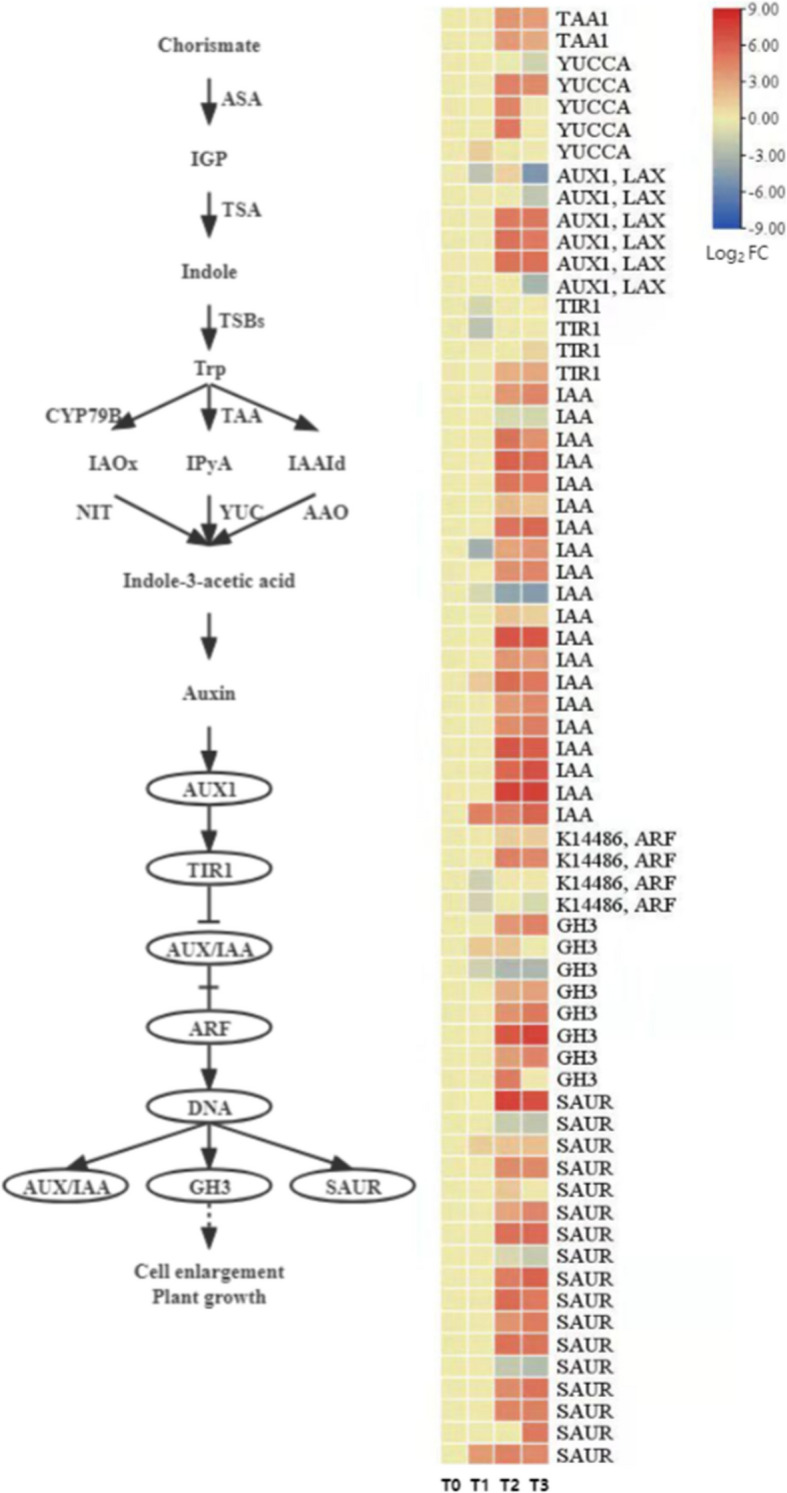
Auxin (IAA) biosynthesis and signaling pathway in *P. lactiflora* seeds during dormancy release

Indole-3-acetic acid (IAA) is one of the key hormones regulating seed dormancy and germination. It promotes seed germination and growth by regulating processes such as cell expansion and embryo axis elongation. During the dormancy release process of *P. lactiflora* seeds, significant changes were observed in the expression of IAA synthesis and signal transduction-related genes, as shown in Fig. 11.

The synthesis pathways of IAA in plants mainly include three routes: the tryptophan pathway, the indole-3-pyruvic acid (IPyA) pathway, and the indole-3-acetonitrile (IAN) pathway. In this study, only genes related to the IPyA synthesis pathway were identified, indicating that this pathway is the primary IAA synthesis route during dormancy release in *P. lactiflora* seeds. In this pathway, two DEGs encoding IPA dehydrogenase (*TAA*) and five DEGs encoding monooxygenase (*YUCCA*) were identified. The expression of the *TAA* gene was upregulated throughout dormancy release, while three *YUCCA* genes showed significant upregulation at T2, suggesting that IAA synthesis is highly active during this stage and contributes to the initiation of seed germination.

In the IAA signal transduction pathway, six DEGs encoding auxin influx carriers (*AUX1*), four DEGs encoding TIR1/AFB F-box proteins, twenty DEGs encoding AUX/IAA repressors, four DEGs encoding ARF transcription factors, eight DEGs from the GH3 family, and seventeen DEGs from the SAUR family were identified. Among these, three *AUX1* DEGs showed significantly upregulated expression during dormancy release, indicating enhanced IAA uptake by cells. The overall upregulation of *AUX/IAA* and *SAUR* proteins suggests that the IAA response pathway is activated, promoting seed germination. Although the expression of *TIR1* did not show significant changes, its downstream *ARF* and *GH3* genes exhibited different expression patterns at different stages, which may be related to the multi-stage functions of IAA in regulating seed dormancy release.

In summary, during the dormancy release process in *P. lactiflora* seeds, IAA is synthesized in large quantities via the indole-3-pyruvic acid pathway and activates signal transduction pathways, promoting the expression of downstream genes and changes in cell activity, thereby providing essential hormonal regulation for seed germination.

#### Expression pattern analysis of cytokinin (CTK) synthesis and signal transduction-related genes

**Fig. 12:**
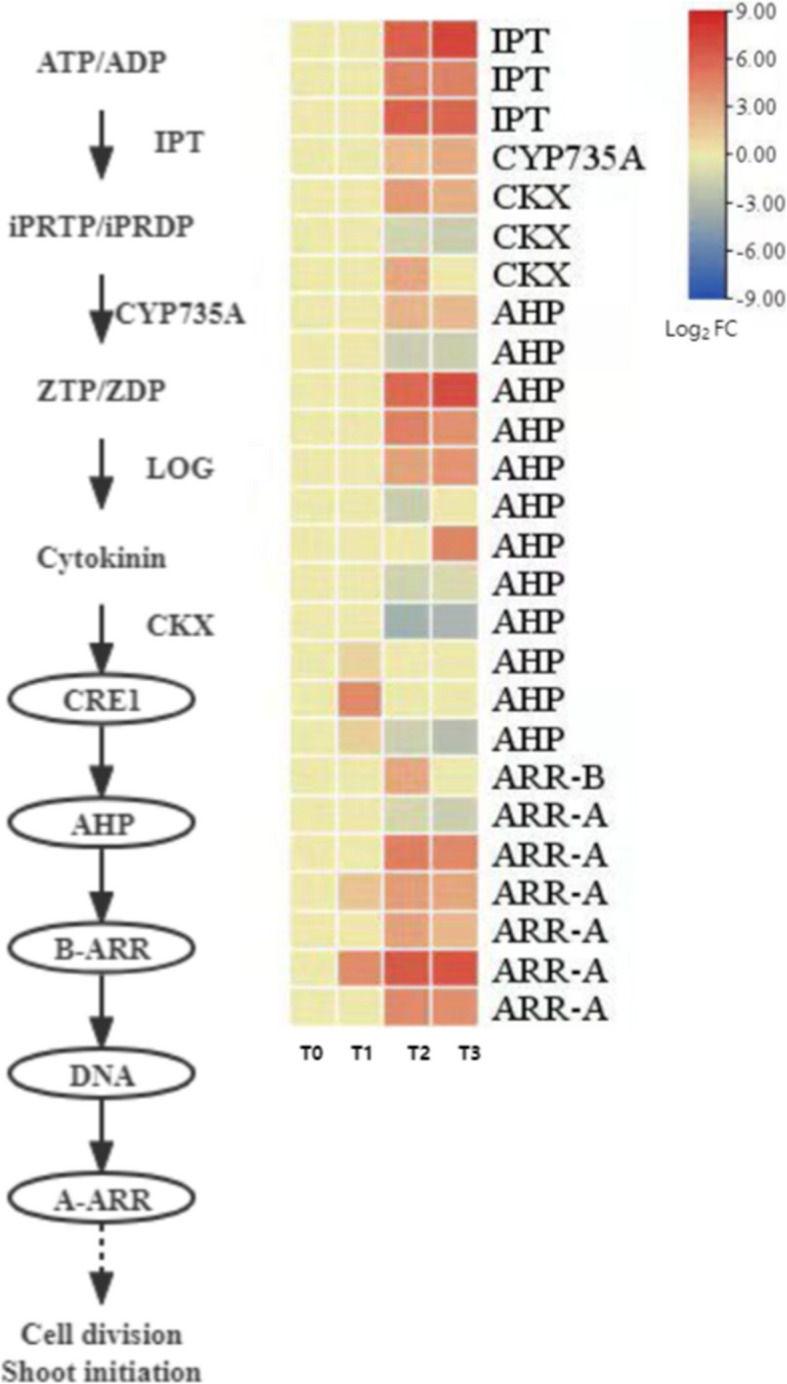
Expression patterns of genes related to cytokinin (CTK) biosynthesis and signaling pathways

Cytokinin (CTK) plays a key regulatory role in seed dormancy release and cotyledon development, primarily by promoting cell division and activating the signal network for bud initiation. During the dormancy release process in *P. lactiflora* seeds, CTK-related synthesis, degradation, and signal transduction genes exhibited stage-specific expression changes (Fig. 12).

In the CTK biosynthesis pathway, this study identified key genes involved in the isopentenyl transferase (IPT) pathway, including three DEGs encoding *IPT* and one DEG encoding cytokinin hydroxylase (*CYP735A*). These genes were generally upregulated during the dormancy release process, suggesting that CTK biosynthesis is particularly active during the early stages of germination. Meanwhile, two of the three cytokinin oxidase/dehydrogenase (*CKX*) DEGs showed significantly increased expression from T1 to T3, which was consistent with the gradual decrease in endogenous CTK content. This suggests that CTK may play a role in maintaining homeostasis during the later stages of dormancy release through its degradation.

In the CTK signal transduction pathway, twelve DEGs encoding histidine phosphotransfer proteins (*AHP*) and seven DEGs encoding two-component response regulators (*ARR*) were identified. The *AHP* proteins act as relay factors, transmitting signals from the CRE1 receptor to downstream *ARR* proteins, thus completing the regulatory signaling chain. The expression patterns of the twelve *AHP* genes varied across the four stages, indicating that different *AHP* members may have stage-specific functions. Among the six type-A *ARR* proteins, five showed significant upregulation during T2 and T3, suggesting that these rapid response factors are active during the later stages of dormancy release, promoting cell division and bud initiation. In contrast, only one type-B *ARR* (transcriptional activator) gene was slightly upregulated at T3, likely involved in initiating transcriptional regulatory responses during the later stages of dormancy release.

In summary, during dormancy release in *P. lactiflora* seeds, CTK regulates bud growth and cell division through both biosynthesis activation and signal response, providing crucial hormonal support for subsequent embryo axis elongation and seed germination.

#### Expression pattern analysis of ethylene (ETH) synthesis and signal transduction-related genes

**Fig. 13:**
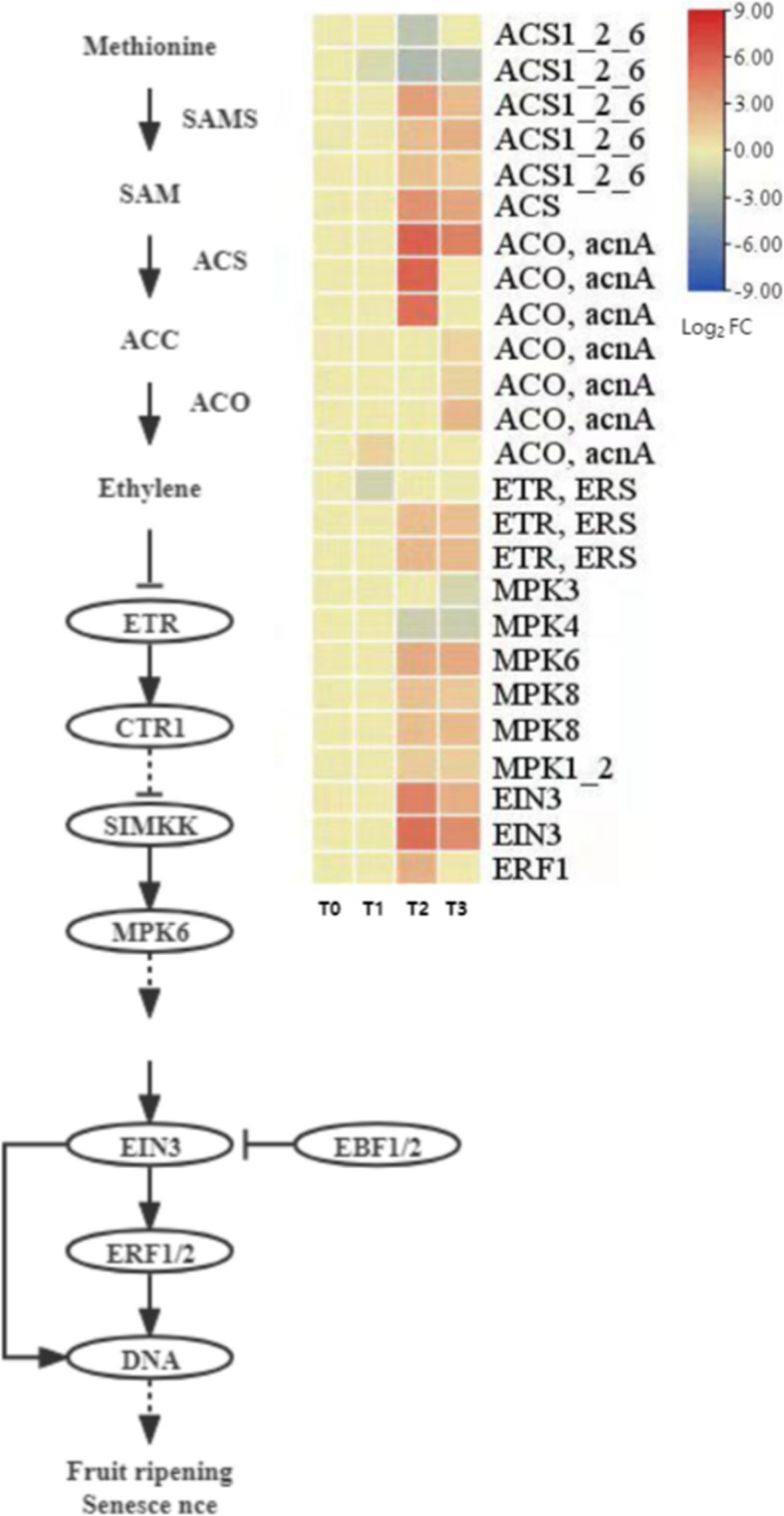
Expression patterns of ethylene synthesis and signaling genes

Ethylene (ETH) is a key hormone that promotes seed germination and embryo axis elongation. It plays a critical regulatory role during the dormancy release process of *P. lactiflora* seeds. This study systematically analyzed the temporal expression patterns of key differentially expressed genes (DEGs) related to ethylene synthesis and signal transduction, as shown in Fig. 13.

In the ethylene biosynthesis pathway, methionine is converted to SAM (S-adenosylmethionine) by SAM synthetase (*SAMS*), which is then converted to ACC (1-aminocyclopropane-1-carboxylate) by ACC synthase (*ACS*), and subsequently catalyzed by ACC oxidase (*ACO*) to produce ethylene. Six *ACS* genes and seven *ACO* genes were identified in this pathway. Of the six *ACS* DEGs, four showed significant upregulation at T2, with their expression peak coinciding with the increase in endogenous ACC levels, suggesting that ACC synthase may be the key rate-limiting enzyme in seed dormancy release. Two other *ACS* genes showed downregulation, but the changes were not significant. *ACO* genes showed a general increase in expression at T2, but the changes were modest throughout, indicating that ethylene production is primarily regulated by *ACS* rather than *ACO*.

In the ethylene signal transduction pathway, three ethylene receptor (*ETR/ERS*) genes, three ethylene insensitive protein 3 (*EIN3*) genes, and six mitogen-activated protein kinase (MAPK) genes were identified. These signaling components exhibited an overall upregulation trend during dormancy release, particularly from T2 to T3. The activation of signal proteins such as *MPK3*, *MPK6*, and *MPK8* may enhance the stability and expression of *EIN3* through phosphorylation cascade reactions, further promoting the transcriptional response of ethylene response factors (*ERF1/2*), and subsequently activating the expression of downstream genes related to cell elongation and seed germination.

In conclusion, during the dormancy release phase of *P. lactiflora* seeds, multiple key genes in the ethylene biosynthesis and signal transduction pathways are activated. This suggests that ethylene enhances signal perception and response, accelerating the breaking of dormancy and the initiation of germination, thus playing a crucial role in regulating the physiological transitions of the seeds.

#### Expression pattern analysis of salicylic acid (SA) synthesis and signal transduction-related genes

**Fig. 14:**
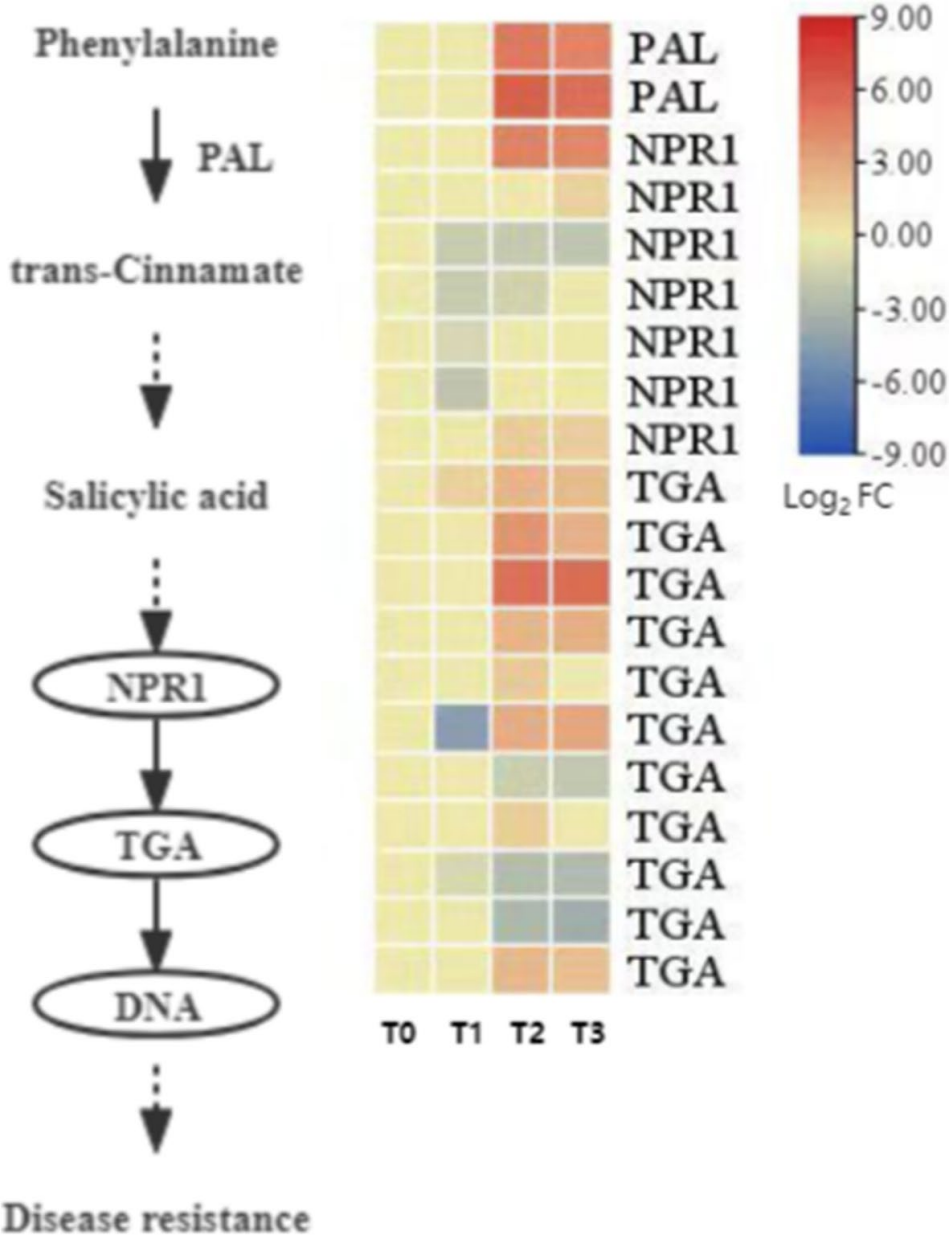
Expression patterns of genes related to salicylic acid synthesis and signaling

Salicylic acid (SA) is a key hormone that regulates plant resistance responses and seed development. During the dormancy release process of *P. lactiflora* seeds, the expression of genes related to SA synthesis and signaling pathways showed specific dynamic changes, as shown in Fig. 14.

In the SA biosynthesis pathway, phenylalanine is catalyzed by phenylalanine ammonia-lyase (*PAL*) to produce cinnamic acid, which is further converted into salicylic acid. In this study, two *PAL* DEGs were identified, both of which exhibited upregulation at all stages of seed dormancy release. This suggests that the synthesis capacity of SA is enhanced during dormancy release. However, the upregulation of *PAL* expression did not fully correlate with changes in endogenous SA content, indicating that there may be differences in transport or metabolic regulation layers.

In the SA signal transduction pathway, seven DEGs encoding non-expressor of pathogenesis-related protein 1 (*NPR1*) and eleven DEGs from the bZIP family of transcription factors (*TGA*) were identified. During dormancy release, the expression of *NPR1* genes generally showed moderate to significant upregulation, and the expression of *TGA* transcription factors also significantly increased at T2 and T3. This suggests that SA may mediate the expression of defense-related genes via the *NPR1-TGA* signaling module and indirectly participate in the physiological transition of seeds.

In summary, during dormancy release in *P. lactiflora* seeds, SA synthesis is enhanced through the upregulation of *PAL* and the activation of the *NPR1-TGA*-dependent signal transduction pathway. This may strengthen the defense response to support the initiation of germination, reflecting the potential developmental role of SA in addition to its role in resistance regulation.

#### Expression pattern analysis of jasmonic acid (JA) synthesis and signal transduction-related genes

**Fig. 15:**
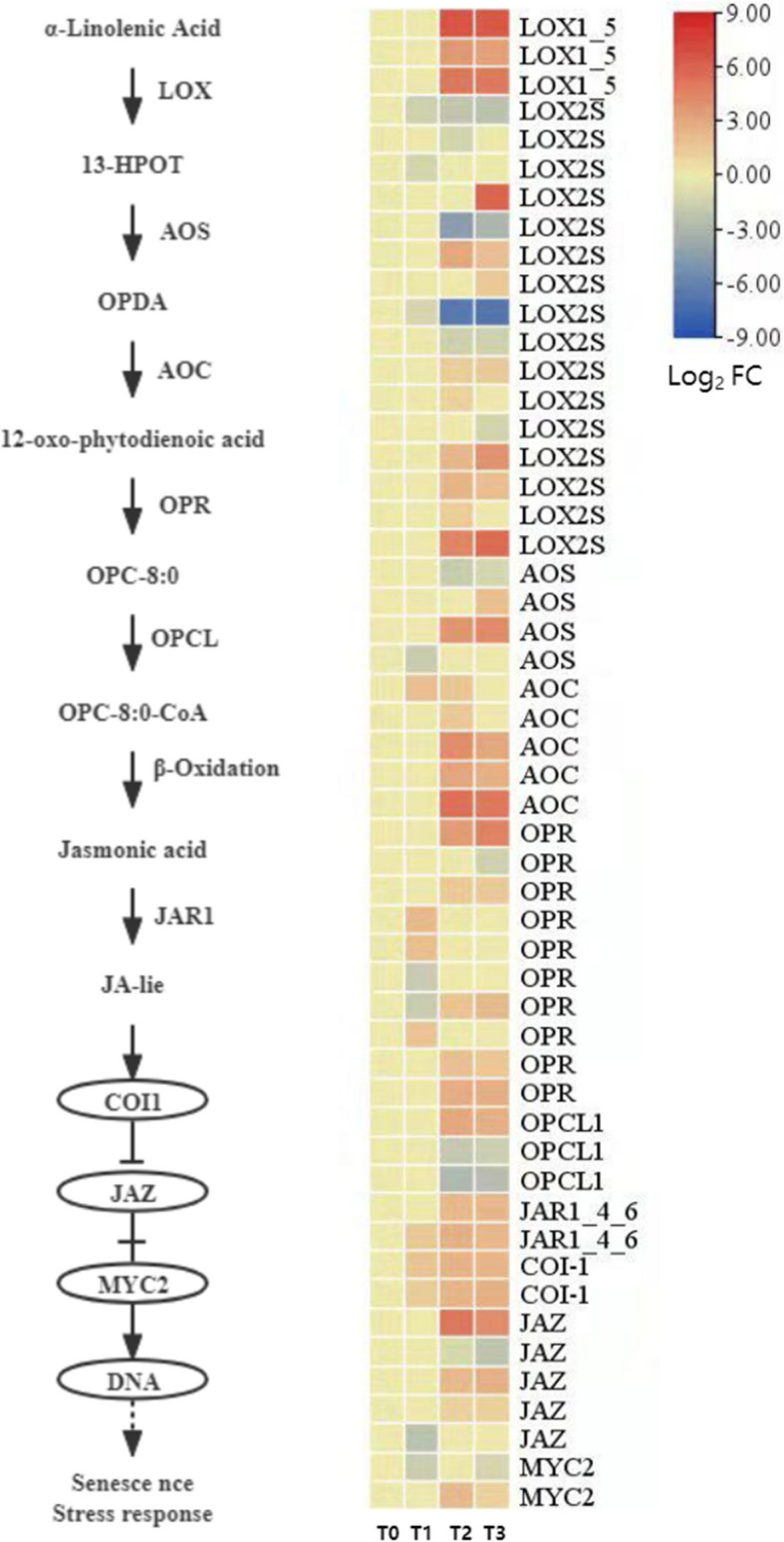
Expression patterns of genes related to jasmonic acid synthesis and signaling

Jasmonic acid (JA) is a key signaling hormone in plants, playing an important role in regulating seed development, senescence, and environmental stress responses. During the dormancy release process of *P. lactiflora* seeds, several genes in the JA biosynthesis and signal transduction pathways were significantly expressed, indicating that JA may play an important regulatory role in this process (Fig. 15).

In the JA biosynthesis pathway, a total of 19 DEGs encoding lipoxygenase (*LOX2S*), 4 DEGs encoding dioxygenase (*AOS*), 5 DEGs encoding cyclase (*AOC*), 9 DEGs encoding 12-oxophytodienoic acid reductase (*OPR*), and 4 DEGs encoding OPC8:0-CoA ligase (*OPCL1*) were identified. *AOC* and *AOS* are key enzymes in the JA biosynthesis pathway, and five *AOC* and four *AOS* genes were significantly upregulated at T2 and T3, which corresponds to the gradual increase in endogenous JA content during dormancy release. Although the expression patterns of the 19 *LOX2S* genes were not entirely consistent across the stages, the overall number and extent of upregulated DEGs were higher than those downregulated. The *OPR* and *OPCL1* family genes exhibited a complex and diverse expression pattern, suggesting they may play different regulatory roles at various stages.

In the JA signal transduction pathway, two *JAR1* (JA-Ile synthetase) genes, two *COI1* (JA receptor E3 ubiquitin ligase) genes, five *JAZ* (JA-ZIM domain protein) genes, and two *MYC2* (bHLH-type transcription factor) genes were identified. The expression of *JAR1* and *COI1* genes showed a general upregulation trend throughout the dormancy release process, suggesting that JA signal response remains continuously active. The expression of *JAZ* and *MYC2* family members showed more variation, with some genes significantly upregulated while others exhibited little change. This indicates that the negative feedback regulation mechanism in the JA signaling pathway may play a crucial role in the seed germination process in *P. lactiflora*.

In summary, the active response of the JA biosynthesis and signal transduction pathways suggests that JA not only participates in regulating the release of dormancy in *P. lactiflora* seeds but may also integrate senescence and stress signaling pathways, providing a regulatory foundation for seed germination.

#### Expression pattern analysis of brassinosteroid (BR) synthesis and signal transduction-related genes

**Fig. 16:**
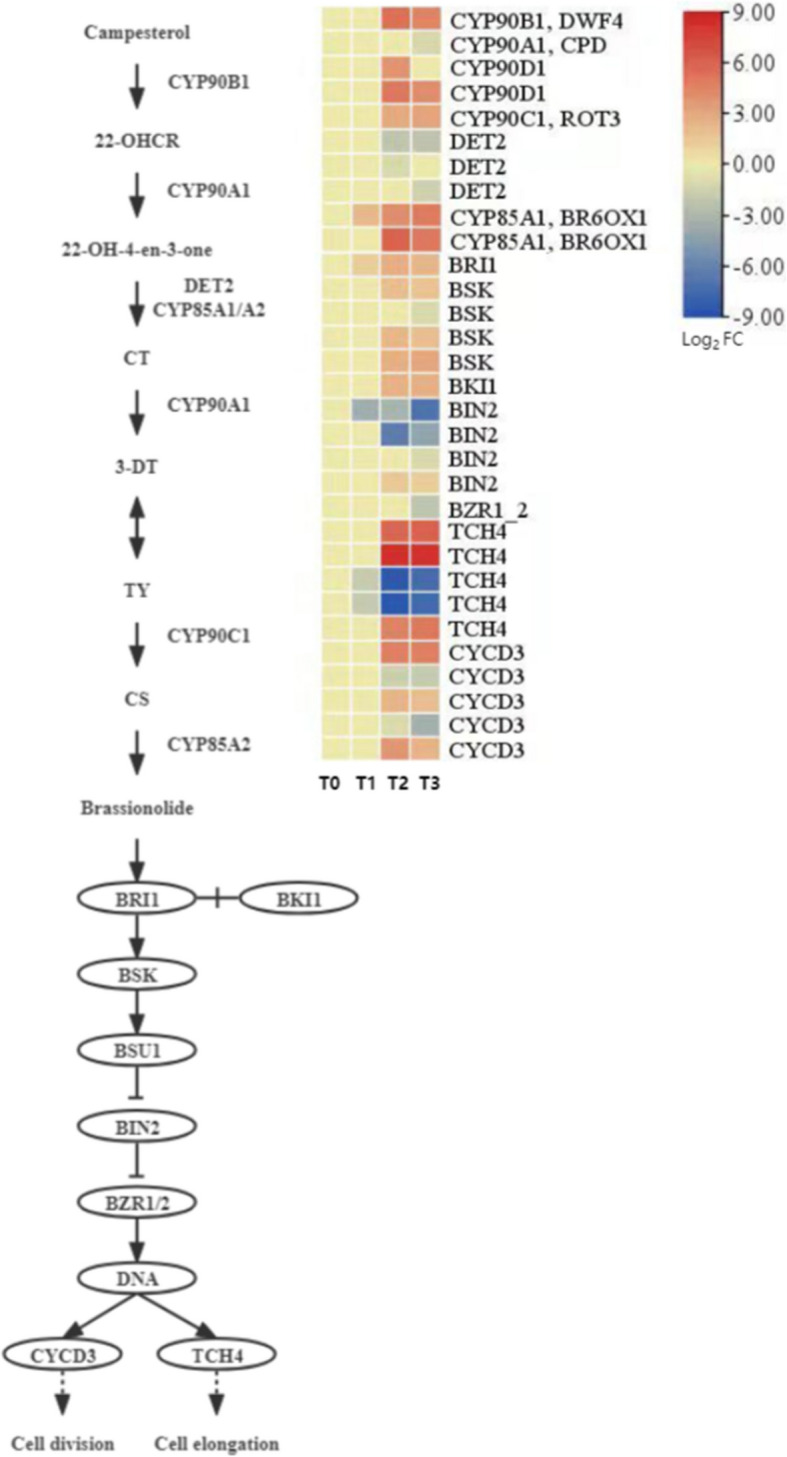
Expression patterns of genes related to brassinosteroid synthesis and signaling

Brassinosteroids (BRs) play a key role in seed development and germination by regulating biological processes such as cell division and elongation. During the dormancy release process of *P. lactiflora* seeds, this study systematically analyzed the expression characteristics of BR synthesis and signal transduction-related genes, as shown in Fig. 16.

In the BR biosynthesis pathway, one DEG encoding BR22-α-hydroxylase (*CYP90B1*), one DEG encoding cytochrome P450 monooxygenase (*CYP90A1*), one DEG encoding C-23 hydroxylase (*CYP90C1*), two *CYP90D1* genes, and two DEGs encoding BR-6-oxidase 1 (*CYP85A1*) were identified. Among these, *CYP90C1*, *CYP85A1*, *CYP90B1*, and *CYP90D1* were generally upregulated during dormancy release, suggesting enhanced BR synthesis activity, which may promote cell division and seed germination. In contrast, the expression of *CYP90A1* did not show significant changes.

In the BR signal transduction pathway, one DEG encoding the BR receptor (*BRI1*), four DEGs encoding BR signal kinases (*BSK*), one DEG encoding the BRI1 kinase inhibitor (*BKI1*), one DEG encoding the BR downstream regulatory factor (*BZR1*), four DEGs encoding BR-insensitive proteins (*BIN2*), five DEGs related to the cell cycle (*CYCD3*), and five DEGs related to cell elongation (*TCH4*) were identified. In *P. lactiflora* seeds during dormancy release, DEGs encoding *BRI1*, *BSK*, *BKI1*, and *CYCD3* were significantly upregulated between T2 and T3, suggesting that they may play a role in promoting cell division and regulating seed germination. Notably, *BZR1* was significantly downregulated at all stages, which may reflect a negative feedback mechanism in the BR signaling pathway. The expression patterns of *BIN2* and *TCH4* were more complex, showing upregulation or downregulation at different stages, indicating that their function may be stage- or tissue-specific.

Overall, the enhanced BR synthesis and activation of certain signaling pathway components suggest that BR plays a positive role in seed dormancy release in *P. lactiflora* by regulating cell proliferation and elongation processes.

### Hormonal regulation mechanisms of dormancy release in *P. lactiflora* seeds during cold stratification

In the cold stratification process of *P. lactiflora* seeds, dormancy release is orchestrated through a series of hormonal and metabolic changes. During T0, at the start of cold stratification, seeds remain in a dormant state, with ABA (abscisic acid) levels remaining high, inhibiting the GA (gibberellin) biosynthesis pathway and IAA (auxin) synthesis, thereby preventing hypocotyl activation. At this stage, hypocotyl activation is not initiated, and low levels of IAA prevent cell division and elongation, maintaining seed dormancy.

As cold stratification progresses to T1 (28 days), ABA levels decrease, initiating GA biosynthesis and releasing DELLA protein inhibition. This results in an increase in IAA levels, activating the IAA signaling pathway and promoting cell division and elongation. Hypocotyl elongation begins, with starch degradation becoming the primary energy source, and α-amylase activity increasing, helping to break down starch into sugars to fuel seed germination.

At T2 (55 days), ABA levels continue to decrease significantly, while GA biosynthesis peaks and IAA levels remain high. The activation of genes like GID1 and PIF4 further promotes hypocotyl elongation, cell expansion, and division. Additionally, acid phosphatase activity increases, suggesting enhanced organic phosphorus metabolism. Starch is converted to sugars, providing the necessary energy for further hypocotyl growth.

**Fig. 17:**
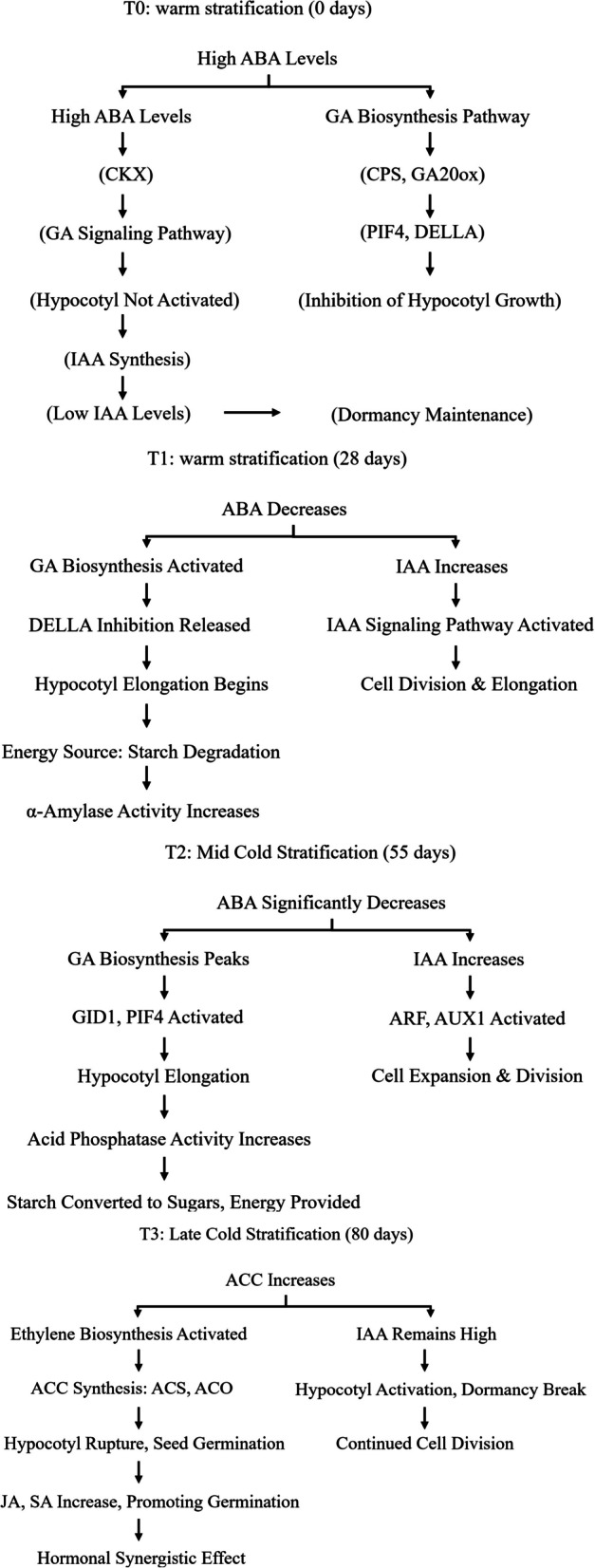
Hormonal Regulation mechanisms of dormancy release in *P. lactiflora* seeds during cold stratification

Finally, at T3 (80 days), ACC levels rise significantly, activating ethylene biosynthesis, which promotes hypocotyl rupture and seed germination. IAA levels remain high, continuing to support cell division and growth. Additionally, JA and SA levels increase, further promoting seed germination. This process demonstrates a complex hormonal synergy involving ABA, GA, IAA, ethylene, JA, and SA, which collectively facilitate the transition from dormancy to seedling emergence (Fig. 17).

### Key gene relative expression analysis in *P. lactiflora*

#### qPCR and RNA-seq correlation validation

In the pathways significantly enriched during the dormancy release process of *P. lactiflora* seeds, 14 genes were selected based on their expression levels and differential expression (q-value < 0.01, FC > 2). These genes include *NCED*, *CYP707A2*, *PP2C*, *KAO*, *GA2ox*, *DELLA*, *YUCCA*, *IPT*, *ACS*, *GPAT*, *BAM*, *AMY*, *PFK*, and *ACSL*. Primers were designed based on the common regions of each gene's transcript.

**Fig. 18:**
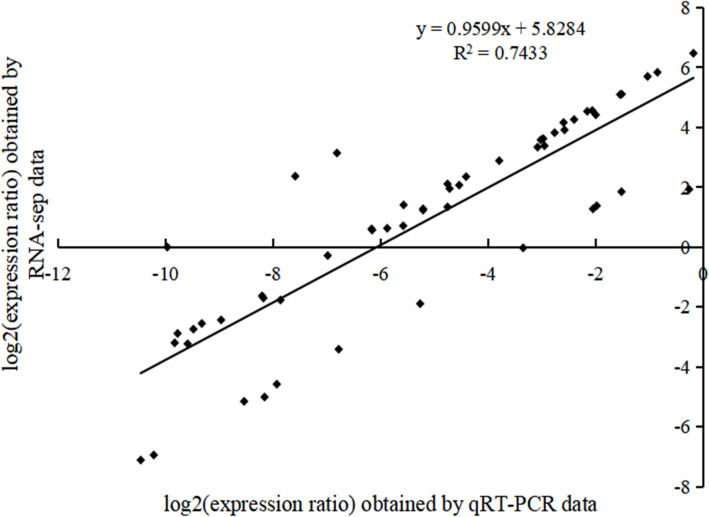
Validation of qPCR and RNA-seq correlation. The figure illustrates the correlation analysis between gene expression ratios obtained by qPCR and RNA-seq. The data show a strong positive correlation (R^2^ = 0.7433), confirming the consistency between the two techniques

The linear regression of the relative expression levels of these 14 genes and the transcriptome FPKM values showed an R^2^ of 0.74, confirming the reliability of the transcriptome data (Fig. 18).

#### Expression analysis of key genes in ABA metabolism

**Fig. 19:**
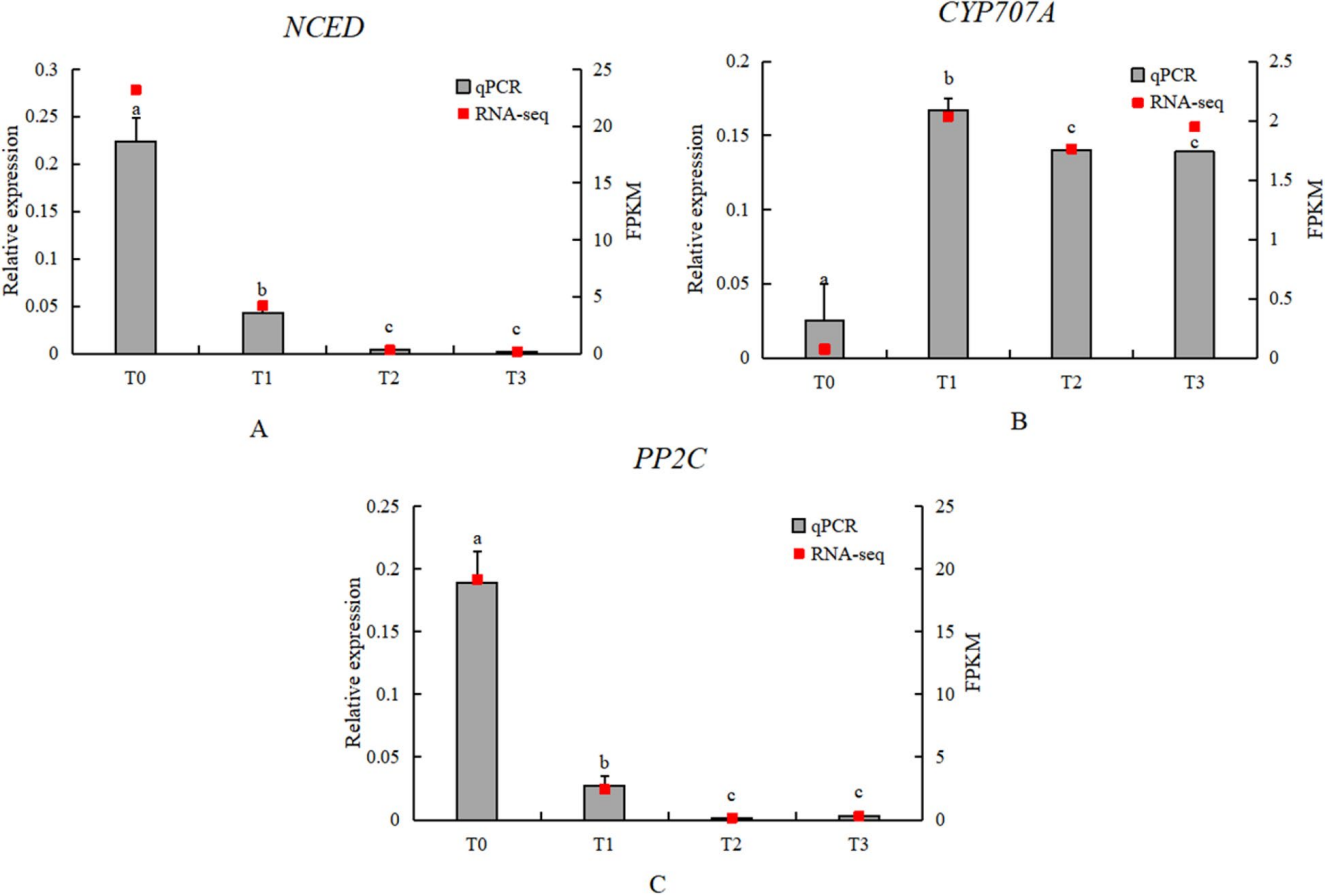
Expression analysis of key ABA metabolic genes (**A**) NCED; (**B**) CYP707A; (**C**) PP2C. Grey bars indicate relative expression levels determined by qRT-PCR (left y-axis), and red squares indicate RNA-seq expression levels (FPKM, right y-axis) across the four stages (T0–T3). Error bars indicate SD. Different lowercase letters above bars indicate significant differences among stages (*P* < 0.05)

In the transcriptome analysis of the ABA metabolism and signal transduction pathways, the average FPKM values of the seven *NCED* transcripts were consistent with the relative expression levels from qRT-PCR analysis. Among the *NCED* transcripts, only one showed continuous downregulation in the transcriptome analysis, while the qRT-PCR results also indicated downregulation of *NCED*, suggesting that only certain gene variants may be involved in the process. Similarly, the transcriptome and qPCR results for *CYP707A* and *PP2C* were consistent (Fig. 19).

#### Expression analysis of key genes in GA metabolism

**Fig. 20:**
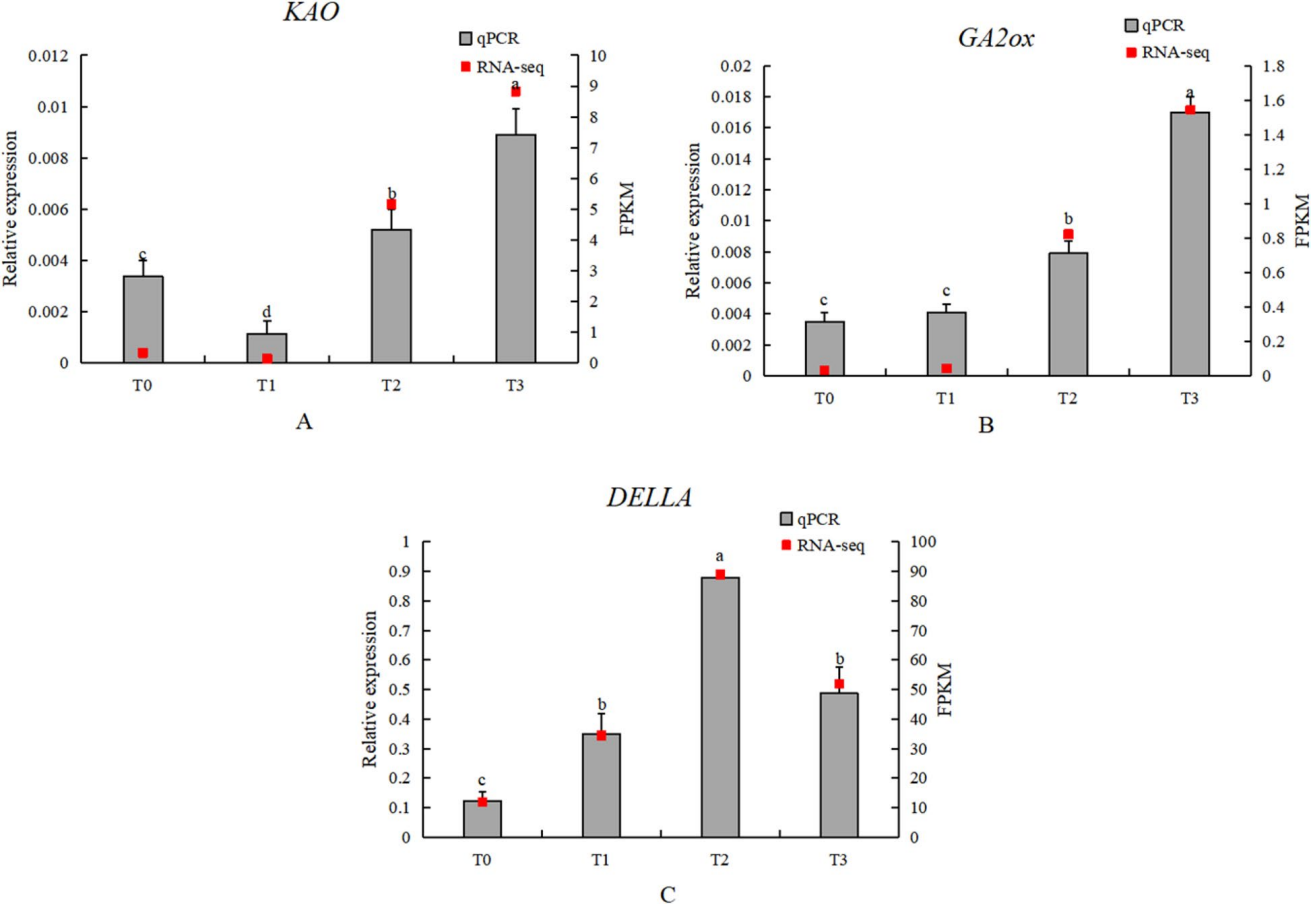
Expression analysis of key GA metabolic genes (**A**) KAO; (**B**) GA2ox; (**C**) DELLA. Grey bars indicate relative expression levels determined by qRT-PCR (left y-axis), and red squares indicate RNA-seq expression levels (FPKM, right y-axis) across the four stages (T0–T3). Error bars indicate SD. Different lowercase letters above bars indicate significant differences among stages (*P* < 0.05)

In the GA biosynthesis, metabolism, and signal transduction pathways, the transcriptome analysis results for *KAO*, *GA2ox*, and qPCR analysis showed some discrepancies in the first two periods, but were consistent in the last two periods. The results for *DELLA* from transcriptome analysis and qPCR analysis were in agreement (Fig. 20).

#### Expression analysis of key genes in IAA, CTK, and ACC metabolism

**Fig. 21:**
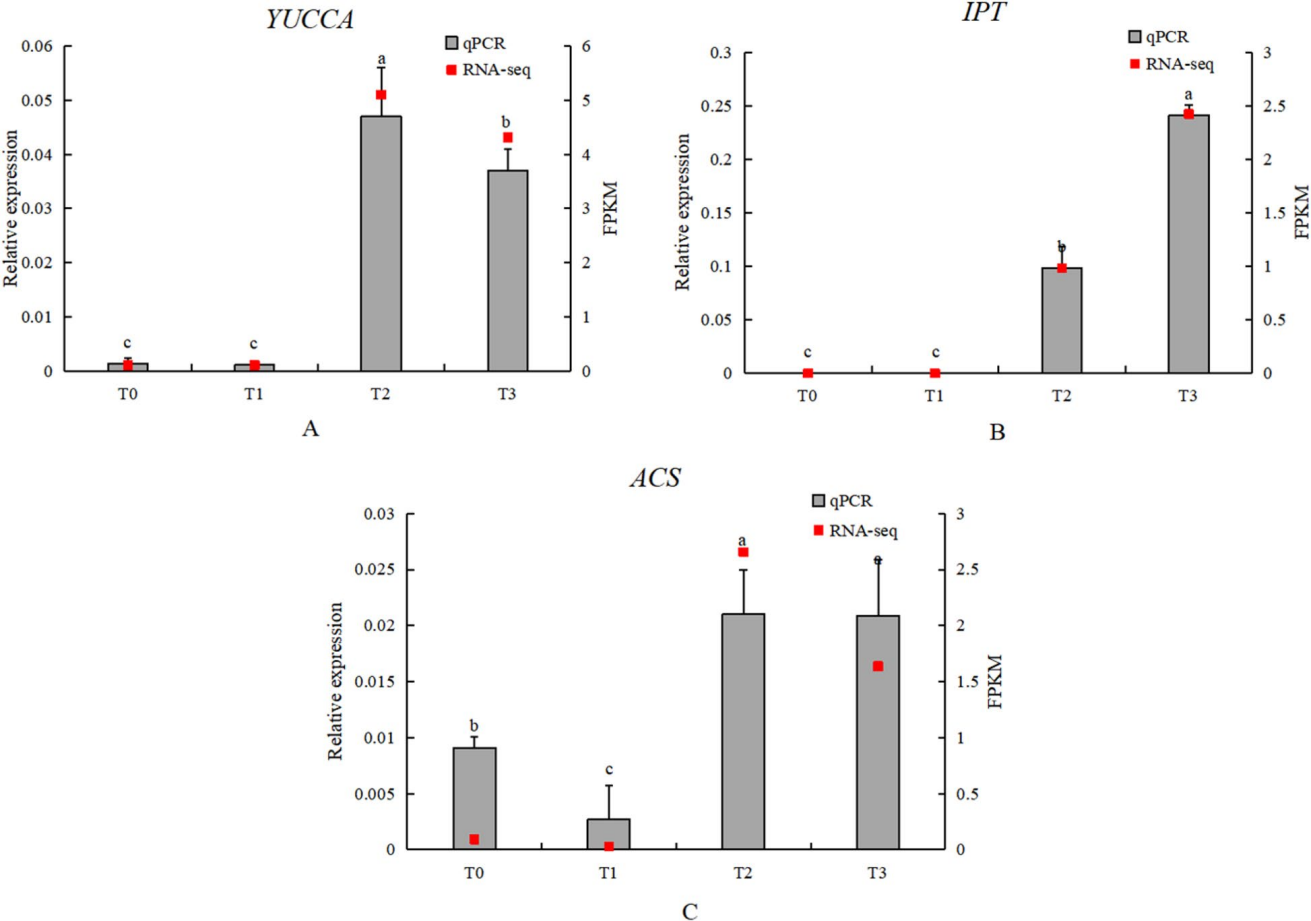
Expression analysis of key IAA, CTK, and ACC metabolic genes (**A**) YUCCA (IAA-related); (**B**) IPT (CTK-related); (**C**) ACS (ACC-related). Grey bars indicate relative expression levels determined by qRT-PCR (left y-axis), and red squares indicate RNA-seq expression levels (FPKM, right y-axis) across the four stages (T0–T3). Error bars indicate SD. Different lowercase letters above bars indicate significant differences among stages (*P* < 0.05)

In the IAA metabolism pathway, the transcriptome analysis results for *YUCCA* were consistent with the qPCR analysis. In the CTK biosynthesis pathway, the *IPT* gene and the *ACS* gene involved in ACC metabolism showed some discrepancies between the transcriptome analysis and qPCR results, although the overall trend of changes was consistent (Fig. 21).

#### Analysis of key genes in carbohydrate and lipid metabolism

**Fig. 22:**
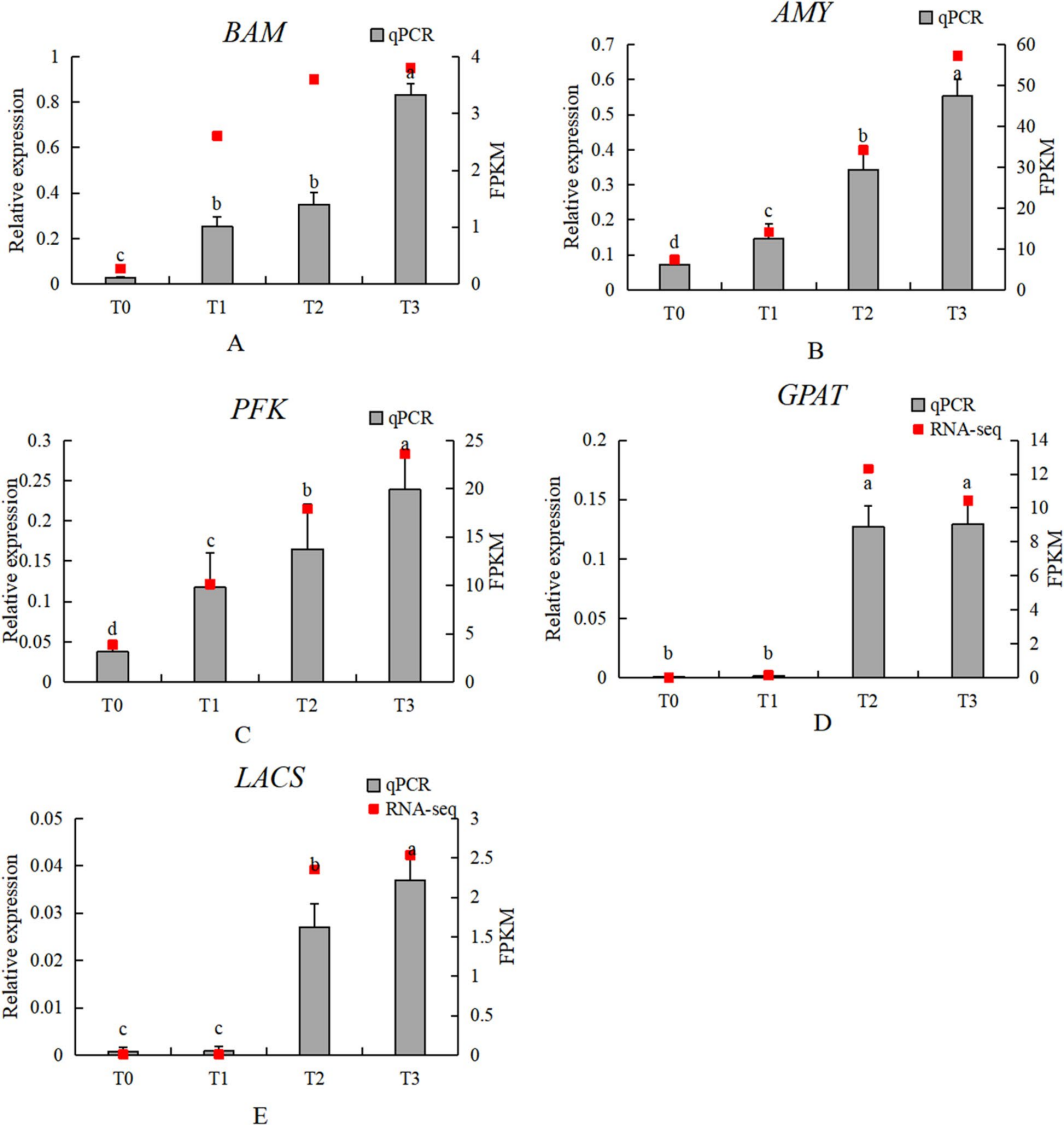
Analysis of key genes in sugar and lipid metabolism (**A**) BAM; (**B**) AMY; (**C**) PFK; (**D**) GPAT; (**E**) LACS. Grey bars indicate relative expression levels determined by qRT-PCR (left y-axis), and red squares indicate RNA-seq expression levels (FPKM, right y-axis) across the four stages (T0–T3). Error bars indicate SD. Different lowercase letters above bars indicate significant differences among stages (*P* < 0.05)

In the lipid degradation pathway, the expression patterns of *ACSL* and *GPAT* differed between the transcriptome analysis and qPCR results. However, the qPCR analysis showed overall upregulation of *ACSL* and *GPAT*, which is consistent with the changes observed during the dormancy release process. For starch and sugar metabolism, the transcriptome and qPCR analysis results for *BAM* were consistent. The expression patterns of *AMY* and *PFK* during *P. lactiflora* seed dormancy release showed differences, with qPCR results for both *AMY* and *PFK* consistently upregulated (Fig. 22).

## Discussion

### Effects of temperature, nutrient mobilization, and hydrolytic enzyme activity on dormancy release in *P. lactiflora* seeds

In this study, we systematically explored the dynamic changes in temperature, nutrient mobilization, and hydrolytic enzyme activities during the cold stratification process of *P. lactiflora* seeds, and compared the findings with existing literature. First, the impact of temperature on root and seedling emergence in our study was consistent with previous research, with the 20 °C treatment group showing the best rooting and emergence rates. Many studies [6, 10] have indicated that suitable temperatures promote seed germination and break dormancy. However, lower temperatures (5 °C, 10 °C) significantly inhibited root and seedling emergence, suggesting that seeds enter a deep dormancy under these conditions, limiting their rooting ability. Our study further reveals how temperature influences seedling growth and epicotyl development by modulating the dynamic changes in endogenous hormones, especially the balance of ABA, GA, and IAA. This finding suggests that the warm stratification phase (0–45 days, 20 °C) mainly promotes radicle growth, while the cold stratification phase (45–80 days, 4 °C) relieves epicotyl dormancy and facilitates seedling emergence. This perspective is in line with the studies of Li et al. [11] and Haq et al. [12] on the role of temperature in plant growth regulation. However, our unique viewpoint is that low temperatures not only affect root growth but also delay radicle development, impacting epicotyl dormancy release—a factor that has been less explored in existing literature.

Further analysis indicated that during dormancy release, the mobilization of storage compounds (such as starch, lipids, and proteins) and the changes in metabolic enzyme activities played a key role. The significant decrease in starch content, along with the degradation of fructose, lipids, and proteins, reflects how the seed mobilizes its stored energy to support germination. This phenomenon aligns with the study by Bialecka et al. on *Amaranthus caudatus* seeds, suggesting that carbohydrates and lipids are the main energy sources for seeds transitioning from dormancy to growth activation. However, the novelty of our study lies in the fact that the degradation of proteins not only provides nitrogen but may also support radicle growth by supplying essential amino acids. At 55 days, protease activity peaked, a phenomenon that is reported for the first time in *P. lactiflora* seeds, further confirming the importance of protein degradation during dormancy release. This result also matches findings in *Cucumis sativus* seed studies [12], but our research further reveals the ongoing supportive role of protein degradation throughout the cold stratification process for seed germination.

Regarding the dynamic changes in hydrolytic enzyme activities, the increase in α-amylase and β-amylase activities was closely related to seed germination, which was especially evident during the cold stratification phase (45–80 days, 4 °C). This finding aligns with studies on *Chenopodium quinoa* Willd [13] and *Jeffersonia dubia* [14], where these enzymes were found to play crucial roles in seed germination. Notably, we also observed that the activities of acid phosphatase and protease continuously increased during cold stratification, a change that is less frequently mentioned in the existing literature. Acid phosphatase activity peaked at 55 days, suggesting its role in organic phosphorus metabolism and phosphorus supply regulation, providing new theoretical support for how seeds manage phosphorus supply. The increase in protease activity indicates that seeds accelerate protein hydrolysis, further providing amino acids for epicotyl growth. This process may be a key step in the transition from dormancy to growth activation, a mechanism not fully elucidated in previous studies.

In conclusion, our study reveals that the seeds of *P. lactiflora* exhibit significant stage-specific characteristics during the warm–cold stratification process. The warm stratification phase mainly promotes root growth, while the cold stratification phase activates the breakdown of stored materials and the expression of hydrolytic enzymes, providing essential energy and metabolic support for epicotyl growth and seedling emergence. Notably, the T1 phase (28 days) is identified as the key period with the most pronounced hormonal changes, laying the physiological foundation for the embryo axis to break dormancy.

### Hormonal dynamics and their role in dormancy release of *P. lactiflora* seeds during cold stratification

This study revealed the dynamic changes in endogenous hormones during the cold stratification process of *P. lactiflora* seeds and further clarified the important role of multi-hormonal synergistic regulation in seed dormancy release. Through the visualization analysis of the relative proportions of 14 major plant hormones and Principal Component Analysis (PCA), we found significant differences in the hormone composition at different cold stratification stages, providing an important physiological basis for breaking dormancy and completing germination in *P. lactiflora* seeds.

First, during the warm stratification phase (0–45 days, 20 °C), we observed a significant downward trend in ABA levels during cold stratification, consistent with the negative regulatory role of ABA in seed dormancy, as reported in other studies [15]. ABA dominated at T0, indicating its core role in maintaining dormancy. As cold stratification time progressed, especially at T2 (55 days), ABA levels rapidly decreased and remained low, further confirming that ABA degradation is one of the key mechanisms for breaking dormancy in seeds [5]. This finding aligns with the study by Khan et al. [16], which highlighted the important role of ABA degradation in breaking the dormancy of *Bunium persicum* seeds.

In contrast, IAA and GA hormones exhibited an increasing trend during cold stratification, particularly at T1 (28 days). IAA levels significantly increased at T1 and remained high, likely playing a key role in early cell division and differentiation regulation, supporting IAA’s role in promoting cell proliferation during the early stages of seed germination [17]. Similarly, both GA₃ and GA₁ levels significantly increased at T1 and T2, indicating that gibberellins play a synergistic role in promoting radicle elongation and seed germination [18]. Our study is consistent with the research by Tuan et al. [19], which demonstrated the promotive effect of GA on breaking dormancy in cereal seeds, particularly during radicle growth and the seed coat rupture process.

Additionally, cytokinin levels showed different dynamic changes. N6-(Δ^2^-isopentenyl) adenosine (N6-iP) significantly increased at T1, and its adenosine form (iPR) also rose at T1, suggesting that cytokinins play a role in regulating growth during the early dormancy release process [20]. However, the levels of tZR and tZ were highest at T0 and significantly decreased afterward, indicating that different forms of cytokinins may play stage-specific roles in dormancy release, a topic not fully discussed in previous studies.

Regarding stress-related hormones, jasmonic acid (JA) and salicylic acid (SA) exhibited different dynamic regulation during cold stratification. JA-Ile content steadily decreased during cold stratification, but peaked at T2, potentially associated with stress responses and late-stage germination regulation [21]. In contrast, SA levels significantly increased at T1, suggesting that SA might play a role in defense or redox regulation during early dormancy release. This result is consistent with previous studies on the role of SA in plant stress responses [18, 22]. Ethylene precursor ACC was almost undetectable at T0 and T1, but increased significantly at T2 (82.95 ng/g) and reached 95.64 ng/g at T3, indicating that ethylene synthesis is activated in the later stages, possibly facilitating seed germination by promoting epicotyl emergence from the seed coat. This phenomenon is consistent with the study by Corbineau et al. [23], which pointed out the promotive role of ethylene in seed germination, especially during the stage when physical barriers are overcome.

In conclusion, the dynamic changes in hormones during dormancy release in *P. lactiflora* seeds reflect a complex multi-hormonal synergistic regulation network. The hormone combinations at different stages reflect how plants regulate their growth and stress response mechanisms according to environmental changes. During cold stratification, hormones such as ABA, IAA, GA, cytokinins, jasmonic acid, and ethylene play key roles, and their changes exhibit clear stage-specific characteristics. Notably, T1 (28 days) was identified as the key period with the most pronounced hormonal changes, laying the physiological foundation for embryo axis breakthrough and dormancy release.

### Molecular mechanisms of transcriptional dynamics during dormancy release in *P. lactiflora* seeds

In this study, based on transcriptomic data, we identified a large number of differentially expressed genes (DEGs) that exhibited significant expression patterns at various stages of dormancy release in *P. lactiflora* seeds, revealing the complexity of gene regulation during the transition from dormancy to germination. Through transcriptomic sequencing of 12 samples, we obtained high-quality sequence data, with sequencing quality reaching a good standard (Q30 value not less than 92.49%), providing a reliable foundation for subsequent analysis. Our results indicate that *P. lactiflora* seeds exhibit significant transcriptomic differences at various dormancy release stages, particularly during the transition from warm stratification (0–45 days, 20 °C) to cold stratification (45–80 days, 4 °C), during which seeds undergo important transcriptional regulatory changes.

During the warm stratification phase (0–45 days, 20 °C), primarily promoting radicle (hypocotyl) growth, gene expression was dominated by genes involved in dormancy maintenance and basic growth processes, with high expression of ABA-related genes supporting the seed in its dormancy state. However, as the process transitioned into the cold stratification phase (45–80 days, 4 °C), the gene expression profile dramatically changed, especially in the epicotyl growth and embryo development stages, with a marked increase in differentially expressed genes. A total of 11,045 DEGs were identified, with 4,933 upregulated and 6,112 downregulated, demonstrating significant transcriptional regulatory changes in seeds during this phase, particularly in genes related to energy metabolism and hormone regulation.

Compared to previous studies [24], we further revealed the dynamic gene expression patterns between different developmental stages. Specifically, during the cold stratification phase (45–80 days, 4 °C), we identified 10,042 DEGs, of which 4,158 were upregulated and 5,884 were downregulated. These DEGs provide crucial molecular evidence for understanding the transition of *P. lactiflora* seeds from dormancy to growth. A total of 923 genes exhibited significant expression changes across stages, suggesting that these genes may play key roles in the critical process of dormancy release.

To better understand the dynamic changes in gene expression, we performed trend analysis on the DEGs. Through this approach, we identified 20 expression trend modules, six of which showed statistically significant enrichment (P < 0.05). The enriched pathways in these modules were mainly related to metabolic regulation, signal transduction, and genetic information processing, reflecting the multi-layered regulatory mechanisms during dormancy release in *P. lactiflora* seeds. Module 9 and Module 17 were significantly associated with the biosynthesis pathway of phenylpropanoids, starch and sucrose metabolism, glycolysis/gluconeogenesis, and plant hormone signal transduction, indicating that energy metabolism and hormonal regulation play critical roles in the transition from dormancy to germination. This aligns with research on the transcriptome of *Solanum torvum* seeds [25] which also found that metabolic pathways and hormone signaling interact to promote seed development during dormancy release and germination.

Notably, the lipid metabolism pathway was significantly enriched in Module 7, suggesting that the synthesis and degradation of fatty acids may play an important role in seed germination. Fatty acids, as an energy source, could provide essential energy support during the early stages of seed germination, promoting cell division and epicotyl growth [26]. The activation of lipid metabolism may be a key physiological marker for the transition from dormancy to active growth in seeds [27]. Similar findings have been reported in transcriptomic studies of other plant seeds [28], emphasizing the critical role of lipid metabolism in seed germination.

Additionally, the enriched pathways in Module 8 and Module 19 highlighted the importance of cellular signaling and gene expression regulation. These pathways involved zeatin biosynthesis, carotenoid biosynthesis, MAPK signaling, DNA replication, and RNA transport, suggesting that precise regulation of cellular signaling and gene expression is crucial for seed growth during germination. The activation of the MAPK signaling pathway may be related to the seed's adaptation to environmental changes during dormancy release, which aligns with Zhu et al.'s study [24] on the role of MAPK signaling in plant responses to external stresses.

### Molecular mechanisms of dormancy release in *P. lactiflora* seeds: insights from WGCNA analysis

In this study, we utilized WGCNA to identify three key co-expression modules (black, cyan, and turquoise) that are highly correlated with the dormancy release process of *P. lactiflora* seeds. By combining expression heatmaps and network structure analysis, we provided an in-depth interpretation of their dynamic expression patterns and potential biological functions. These findings align closely with existing research on seed dormancy and germination mechanisms in other plant species, and they highlight both the commonalities and unique features of dormancy release in perennial herbaceous plants like *P. lactiflora* under cold stratification conditions.

The *black* module displayed a "late activation" expression pattern, with strong positive correlations in the T2-T3 stages with key germination indicators such as GA₃, IAA, and α-amylase. This suggests that it may serve as a key regulatory unit in embryo activation and metabolic initiation. A similar result was reported in sorghum (*Sorghum bicolor*), where a red module was identified as being associated with late-stage germination, enriched with genes involved in carbohydrate metabolism and cell wall loosening. This module showed significant upregulation during the breaking of seed coats [29]. Additionally, in *Triticum aestivum*, the expression of GA response elements in late germination stages was significantly enhanced, consistent with the dynamics of GA-related hub genes in the *black* module, such as *TRINITY_DN31548_c0_g1_i2_3* [29].

The *cyan* module, with its "early high expression, rapid downregulation later" pattern, was enriched with hub genes related to ABA, JA signaling, and stress response pathways, suggesting its critical role in maintaining dormancy and responding to cold stress. This conclusion is similar to findings from a study on *Astragalus membranaceus* seeds using WGCNA, where the turquoise module, upregulated during early cold stress treatment, enriched genes involved in cold response proteins, ABA negative regulators, and ethylene biosynthesis [30]. In *Amomum tsaoko*, a similar "early activation" of ABA pathway genes, such as PP2C and ABI5, was observed, further supporting the role of the *cyan* module in early dormancy maintenance during cold stratification in *P. lactiflora* seeds [31].

The *turquoise* module, which showed significantly high expression at the T0 stage followed by gradual silencing, appears to primarily focus on early signal reception, maintaining embryo tissue stability, and regulating redox homeostasis. This characteristic aligns with the blue module in *Triticum aestivum*, which was significantly enriched during early dormancy and contained genes related to ROS clearance systems, storage protein, and inhibition of embryo axis development [32]. Furthermore, in *Chenopodium quinoa* seed germination studies using WGCNA, a hub module was found to be highly expressed at the start of dormancy, enriched with antioxidant system-related genes such as CAT and GST, aligning with the annotation of hub genes like *TRINITY_DN21904_c0_g1_i1_2* in our study [33].

It is noteworthy that *P. lactiflora* seeds exhibit typical morphological and physiological dormancy, and their dormancy release is governed by the synergistic effects of cold stratification and endogenous hormone balance. This differs significantly from seeds of other model species like *Phyllostachys edulis*, *Triticum aestivum*, or *Arabidopsis thaliana*. Therefore, the persistent activation of the *cyan* and *turquoise* modules during the early stages of cold stratification in *P. lactiflora* may represent a characteristic "multi-stage response regulatory system" unique to perennial herbaceous plants. The hub genes within these modules exhibit a time-dependent hierarchical structure of "signal reception–transcription stabilization–metabolic activation," a feature that has not been systematically elucidated in studies of perennial plant seeds.

In summary, the *black*, *cyan*, and *turquoise* modules share functional characteristics and expression dynamics that align with findings from other plant studies, validating the general applicability and effectiveness of WGCNA in analyzing seed germination mechanisms. The unique physiological dormancy release characteristics of *P. lactiflora* also impart more complex regulatory layers and biological significance to these modules, providing important insights and comparative perspectives for understanding the dynamic transcriptional regulation of perennial plant seed dormancy and germination.

### The role of plant hormones in the dormancy release of *P. lactiflora* seeds

In this study, we systematically analyzed the expression of hormone metabolism-related genes during the cold stratification process in *P. lactiflora* seeds, revealing how the dynamic changes in hormones such as ABA, GA, IAA, CTK, ETH, SA, JA, and BR coordinate at different stages to drive the transition from dormancy to germination. Our results suggest that the interaction and regulation of plant hormones play a crucial role in dormancy release in *P. lactiflora* seeds. This finding provides a new perspective for understanding the molecular mechanisms underlying seed development in *P. lactiflora*.

First, during the warm stratification phase (0–45 days, 20 °C), we observed that ABA (abscisic acid), as a core regulatory factor for seed dormancy, exhibited a significant decline during cold stratification. The rapid degradation of ABA plays a key role in dormancy release, supporting the close relationship between ABA degradation and dormancy release. We found that the ABA biosynthesis gene *NCED* was significantly upregulated in the early stage (from T0 to T3), while ABA-metabolism-related CYP707A genes showed increased expression in the later stages, which correlates with the decline in ABA content. This result is consistent with previous studies, confirming that ABA degradation is one of the key mechanisms for breaking seed dormancy [34]. According to Xu et al. [35], ABA regulates water balance, inhibits cell division and expansion, thereby maintaining seed dormancy. Our study further emphasizes the dynamic balance between ABA synthesis and metabolism in dormancy release, providing new molecular evidence.

During the cold stratification phase (45–80 days, 4 °C), we found that the levels of GA (gibberellins) significantly increased in the early cold stratification stage (T1 and T2), especially GA₃, which played a key role in promoting radicle elongation and seed germination. The activation of GA synthesis contrasts with ABA degradation, suggesting the importance of the interaction between GA and ABA in dormancy release. This finding aligns with Zhao et al. (2017) [36], which discussed the role of GA in breaking seed dormancy, where GA interacts with DELLA proteins to relieve the inhibition of epicotyl growth, thus promoting seed germination. We further revealed that the enhancement of GA synthesis and the downregulation of GA2ox jointly maintain the biological activity of GA during seed germination, providing molecular support for understanding the role of GA in seed germination.

IAA (auxin) also played an important role in the dormancy release process of *P. lactiflora* seeds. Our study showed that during cold stratification, IAA synthesis significantly increased at T1 and remained at a high level at T2 and T3. This is consistent with the role of IAA in cell division and expansion, suggesting that IAA may promote radicle elongation and seed germination by regulating cell growth and division. We found that the primary pathway for IAA biosynthesis was the indole-3-pyruvic acid (IPyA) pathway, which aligns with the findings of Jayasinghege et al. in their study of *Pisum sativum* [37]. IAA signaling-related genes, such as AUX1, TIR1, and SAUR, showed different expression patterns during dormancy release, further proving the dynamic regulatory role of IAA in dormancy release and epicotyl development.

Regarding cytokinins (CTK), our research found that CTK biosynthesis was more active during the early dormancy release phase (T1) and gradually decreased as cold stratification progressed. This suggests that CTK may participate in the later-stage homeostatic regulation by degradation. Our results indicate that the dynamic changes in cytokinin levels are closely related to epicotyl division and growth, which is consistent with the study by Xu et al. [35] on the role of CTK in epicotyl development in *Triticum aestivum*. By analyzing the expression of AHP and ARR genes, we found that cytokinin signaling was activated from T2 to T3, suggesting that CTK may support seed germination by promoting cell division and proliferation.

The role of ethylene (ETH) in dormancy release in *P. lactiflora* seeds was also confirmed in our study. We observed that the ethylene precursor ACC gradually increased during cold stratification, particularly at T2, indicating that ethylene signaling plays an active role in the embryo breaking through the seed coat. Ethylene activates relevant signaling pathways, particularly the upregulation of MPK3 and MPK6, enhancing the seed’s response to environmental changes, thus promoting seed growth and germination. This result aligns with Wang et al. [38], who discussed the role of ethylene in seed germination, further validating the critical role of ethylene in breaking seed dormancy.

Regarding salicylic acid (SA) and jasmonic acid (JA), although their primary roles differ, both play key regulatory roles during seed dormancy release. SA significantly increased during the early cold stratification phase, possibly supporting dormancy release by regulating seed resistance, while JA plays a role in stress response and late-stage seed germination. Our findings indicate that the dynamic changes in SA and JA signaling suggest they not only play a role in plant stress tolerance but also may play an auxiliary role in seed germination and growth. This is consistent with previous studies on the role of SA and JA in seed development [28]. The synthesis and signaling of brassinosteroids (BR) also exhibited significant changes, particularly in the later stages of seed dormancy release. We found that BR synthesis and signaling were significantly activated from T2 to T3, suggesting that BR plays a role in cell division and elongation. The activation of BR may support the seed germination process by regulating cell proliferation and elongation. This finding aligns with Zhong et al. [39], who studied *Arabidopsis*, indicating that BR promotes seed germination by regulating cell wall synthesis and cell division.

## Conclusion

This study provides a detailed understanding of the dynamic changes in endogenous hormones and their role in dormancy release in *P. lactiflora* seeds during warm and cold stratification. Through the analysis of hormone regulation and transcriptomics at different stages of cold stratification, we found that the warm stratification phase (0–45 days, 20 °C) primarily promoted radicle growth, with ABA (abscisic acid) playing a dominant role in maintaining dormancy. As cold stratification commenced, the cold stratification phase (45–80 days, 4 °C) induced more complex hormonal regulation, with significant increases in IAA (indole-3-acetic acid) and GA (gibberellin) levels, alongside rapid degradation of ABA, facilitating epicotyl growth and seed germination.

During the warm stratification phase, ABA synthesis and metabolism played a crucial role in maintaining dormancy. As the process transitioned to cold stratification, the dynamic hormonal changes reflected the physiological shift from dormancy to germination. Specifically, in T2 (55 days), the significant increases in GA and IAA provided important hormonal support for epicotyl elongation and seedling emergence. Through transcriptomic analysis, we identified 11,045 differentially expressed genes (DEGs), which highlighted significant gene expression changes during the cold stratification phase (T0–T3), providing a molecular foundation for the transition from dormancy to germination.

Moreover, WGCNA analysis revealed the regulatory roles of the black, cyan, and turquoise modules at different warm and cold stratification stages, reflecting the key roles of hormone metabolism and energy mobilization during dormancy release. Specifically, the cyan module was closely related to ABA and JA signaling regulation in the early cold stratification phase, possibly playing a significant role in cold stress response and dormancy maintenance. In contrast, the turquoise module was associated with early signal reception, embryo tissue stability, and redox homeostasis regulation, indicating its important function in dormancy release.

In conclusion, the warm stratification phase provided initial support for root growth in *P. lactiflora* seeds, while the cold stratification phase relieved epicotyl dormancy and facilitated the transition from dormancy to growth activation. Through the alternating warm and cold stratification, the seeds underwent dynamic hormonal changes, mobilization of stored nutrients, and activation of hydrolytic enzymes, successfully transitioning from dormancy to germination. Our findings offer new insights into the dormancy release mechanism in perennial plants and provide a theoretical basis for optimizing seed propagation and cultivation techniques.

## Supplementary Information

Additional file 1. Primers for qRT-PCR validation of transcriptiome data

## Data Availability

All datasets generated and analyzed in this study have been deposited in NCBI and are publicly available under BioProject accession PRJNA1312232 ([http://www.ncbi.nlm.nih.gov/bioproject/1312232]).

## Abbreviations

ABA: Abscisic Acid
GA₃: Gibberellic Acid
IAA: Indole-3-Acetic Acid
DEG: Differentially Expressed Gene
WGCNA: Weighted Gene Co-expression Network Analysis
HPLC-MS: High-Performance Liquid Chromatography-Mass Spectrometry
FPKM: Fragments Per Kilobase of Transcript Per Million Mapped Reads
PCA: Principal Component Analysis
KEGG: Kyoto Encyclopedia of Genes and Genomes
qRT-PCR: Quantitative Real-Time Polymerase Chain Reaction
TCA: Tricarboxylic Acid Cycle
MAPK: Mitogen-Activated Protein Kinase
RNA-seq: RNA Sequencing
