# Supplementary material for: Molecular mechanisms of seed dormancy release in Paeonia lactiflora revealed through transcriptomic and metabolomic analysis
Source: BMC Plant Biol. 2026 Apr 16;26:702. doi: 10.1186/s12870-025-07636-x (PMC13088584; doi:10.1186/s12870-025-07636-x)
Supplement: Supplementary file 1 — Additional file 1. Primers for qRT-PCR validation of transcriptiome data [file 12870_2025_7636_MOESM1_ESM.docx]

Additional Files

Additional file 1 — Primers for qRT-PCR validation of transcriptiome data

| Num | Gene Symbol | Forward primer (5->3) | Reverse primer (5->3) |
| --- | --- | --- | --- |
| 1 | *NCED（TRINITY_DN24231_c0_g4_i1_2）* | TGTTCCCGAATGTCCTGT | TTCGCTCCGTTACGAAGA |
| 2 | *CYP707A2 （TRINITY_DN28154_c0_g1_i1_2）* | GGTGTGGCTGTATTGTCTATCT | GAAAGGAATTGTAACCTCCGTC |
| 3 | *PP2C（TRINITY_DN23662_c1_g1_i1_1）* | TGGTGTTGGTGGGAAAGAA | GACTTTGCCACCGCGATA |
| 4 | *Kao (TRINITY_DN33870_c0_g4_i9_3）* | ATCTGTGGAGTGACTCTCTT | CGTGATATTGATGGGAGCTG |
| 5 | *GA2ox（TRINITY_DN20997_c0_g1_i1_3）* | CTCCTGACCCTACTGACTT | GGGCTTGGTTGAGTTTGAT |
| 6 | *DELLA（TRINITY_DN34641_c0_g1_i1_2）* | TCCTCTGACATGGTTCACTAC | AGTGTCAATCGGAGGAGC |
| 7 | *YUCCA（TRINITY_DN32916_c0_g1_i1_4）* | CTGCAACAGAGGTGGTATG | TTCCACTTCCTTGAAGAAGTCC |
| 8 | *IPT（TRINITY_DN19933_c0_g1_i1_3）* | AAGGACTCAACATAGTCACCA | GGATCAGCCATACCTAGCAA |
| 9 | *ACS（TRINITY_DN40231_c0_g1_i1_3）* | TCCGCGAAATTATGTATTCTGG | GGTTGGTTTGGAGTGTGG |
| 10 | *BAM（TRINITY_DN33870_c0_g4_i9_3）* | CAATGGGTGACAGAAATTGG | ATGAGAGGCATTTAGGATTGTG |
| 11 | *AMY（TRINITY_DN40755_c2_g1_i4_2）* | GCAGCTTACTGTTGGATGTAG | GTTGATCCCATTCACTGCC |
| 12 | *PFK（TRINITY_DN25148_c0_g1_i3_3）* | GTCGAATCTCTGTTCCTGAAG | CCTCACCGAAACTAAACCT |
| 13 | *GPAT (TRINITY_DN29299_c0_g1_i4_4)* | CAGAGCTATCAGAACAGACC | GCTTCAAAGGCTACAAGCAT |
| 14 | *LACS (TRINITY_DN23766_c0_g1_i1_4)* | CATGCAGAGGTTGATTTCG | TTTCAGCCGTTGAGCAGACA |
